# Genomic and Transcriptomic Profiling of Amino Acid Compositions in Common Carp Fillets

**DOI:** 10.3390/ani15091335

**Published:** 2025-05-06

**Authors:** Yingjie Chen, Kaikuo Wang, Qi Wang, Yiming Cao, Ran Zhao, Yan Zhang, Jiongtang Li

**Affiliations:** 1 National Demonstration Center for Experimental Fisheries Science Education, Shanghai Ocean University, Shanghai 201306, China; cyjttkl@163.com (Y.C.); kkwangbj@163.com (K.W.); 2 Key Laboratory of Aquatic Genomics, Ministry of Agriculture and Rural Affairs, Beijing Key Laboratory of Fishery Biotechnology, Chinese Academy of Fishery Sciences, Beijing 100141, China; qiwang1993@gmail.com (Q.W.); caoyiming@cafs.ac.cn (Y.C.); zhaoran@cafs.ac.cn (R.Z.); zhangy@cafs.ac.cn (Y.Z.)

**Keywords:** amino acids, common carp, genome-wide association study, transcriptome

## Abstract

Fish are rich sources of amino acids (AAs), particularly human essential amino acids (HEAAs). Exploring the regulatory mechanisms behind the changes in the combined AA content in the fillet and enhancing the content of AAs, especially HEAAs, in fillets of farmed fish is crucial for meeting human nutritional needs. After hot acidic hydrolysis of 304 common carp fillets, we quantified the contents of 17 single AAs and 5 AA groups and observed significant variations among them. Except for Pro, 16 single AAs and all AA groups showed medium-to-high heritabilities over 0.2. Through a genome-wide association study (GWAS), we identified 1974 SNPs and candidate genes associated with at least one AA content. Using transcriptome data from groups with the highest and lowest contents for each AA, 7089 candidate genes were related to the concentrations of at least two AAs. For the total HEAA content, 121 SNPs and their associated genes preferred ATPase-coupled transmembrane transporter activity, and 4727 differentially expressed genes were enriched in cytokine activity, chemokine activity, oxidoreductase activity, and ion binding. With the optimal genomic selection programs and associated SNPs, the correlation between the actual AA contents and estimated breeding values was high and positive, ranging from 0.76 to 0.90. These findings revealed the major-effect processes and regulatory mechanisms modulating the differences in fillet AA contents. The genomic selection programs will guide the future selection of common carp with high AA contents.

## Simple Summary

Farmed fish are an important source of protein and provide human essential amino acids (HEAAs). Characterizing the variation in combined amino acid (AA) composition in fish fillets and exploring the mechanisms regulating variations is essential for advancing breeding strategies and enhancing fillet AA levels. Although numerous studies have focused on AA metabolic processes, the regulatory mechanisms behind the content variations remain elusive. Herein, we quantified the fillet AA contents of 304 farmed common carp samples with acidic hydrolysis and conducted a genome-wide association study and RNA-seq analysis. This study aims to elucidate the genetic variations and differential expressions underlying the fillet AA contents. Our findings showed high variations in the fillet AA contents among these samples. Through a genome-wide association study, the SNPs and candidate genes associated with AA content were identified. The differential expressions also possibly diversify the fillet AA contents. For the total content of HEAA, the SNPs and associated genes were involved in transporter activity, while the differentially expressed genes preferred cytokine binding, oxidoreductase activity, and ion binding. Our findings not only provide new insights into the molecular mechanisms underlying the fillet AA contents but also support developing a selection strategy to improve fillet AA levels in common carp.

## 1. Introduction

Amino acids (AAs) are widely used in the human food industry, crop farming, livestock farming, and aquaculture. Unlike free AAs in animal serum, combined AAs in proteins are the primary nutrient components that determine flesh quality. To satisfy human nutrient demand, AAs are categorized as essential and non-essential, with essential AAs needing to be obtained through diet. The fillets of some fish contain all the human essential amino acids (HEAAs), which are combined in the proteins [1]. The HEAA content in fish fillets was higher than that in meat [2]. Therefore, increasing the content of AAs, especially HEAAs, in fish fillets is of great significance for human nutritional demand.

Two strategies have been proposed to elevate the combined amino acid (AA) content in fish fillets: supplementing fish feed with exogenous amino acids and enhancing the fish’s ability to synthesize endogenous amino acids. Protein represents the costliest nutrient in fish feeds, and its inclusion levels generally fall within the range of 30~50% [3]. To fulfill the nutritional requirements of humans and fish, fish meal has long been utilized as the principal protein source in feeds, mainly due to its high-quality protein content and meticulously balanced AA profile [4]. However, given that the production of fish meal is limited while it is in high demand in aquaculture, as well as livestock and poultry farming, it is challenging for this strategy to satisfy the growing need for AAs. Augmenting the endogenous synthesis capacity of AAs or reducing AA consumption in fish can significantly diminish reliance on exogenous fish meal. Concurrently, it can enhance the quality of fish fillets and bestow health-promoting effects. Hence, breeding fish with improved fillet AA content would be beneficial to human dietary nutrition, farming, and aquaculture. Nevertheless, research efforts to identify genetic variants capable of modulating amino acid metabolism and formulate breeding strategies remain scarce.

In soybean [5,6], bread wheat [7], maize [8], duck [9], and rainbow trout [10], SNPs have been identified as significantly associated with AA composition. The AA metabolism encompasses a series of intricate biological processes, including transportation, synthesis, and catabolism. The AA uptake by cells or organelles requires the AA transporters (AATs) [11]. The ATP-binding cassette (ABC) transporter and the solute carrier (SLC) superfamily are the two largest components of AATs [12,13]. AATs transport amino acids through ion conjugation [14]. The non-essential AAs can be synthesized through different biosynthetic enzymes [15]. The AAs were utilized for protein synthesis and generating nitrogenous compounds, or oxidized as an energy source [16]. Given that numerous metabolic pathways are intricately intertwined in AA metabolism, the key pathways governing the differences in the fillet AA composition and content still remain elusive and have not been comprehensively studied. Unraveling the major-effect genes and metabolic pathways, and further clarifying their regulatory mechanisms on the disparities in AA concentrations, will offer novel directions to boost the AA content of fish fillets.

Common carp (*Cyprinus carpio*) is a freshwater fish species that is widely cultivated around the world, and various strains have been cultured for thousands of years [17]. It is estimated that this fish accounts for approximately 10% of global freshwater aquaculture production [18]. If AA consumption per sample decreased, it would be significant in reducing the pressure of AA production. Considering the huge feed consumption and distinct nutrient value of common carp, it is crucial to conduct extensive research to comprehensively understand the metabolism patterns of AA content in its fillets. The AA content varied significantly among individual common carp [19]. Using a 250 K SNP array, Zhang et al. identified thirty-six, six, and one SNPs associated with Gly, Pro, and Tyr content. Due to the low genome coverage of this SNP array, the existence of more genetic variants associated with the contents of these three amino acids (AAs), as well as those associated with other AAs, remains unknown and has not been fully explored. Here, we integrated genome and transcriptome analyses to identify key genes and primary metabolic pathways, and clarify their regulatory mechanisms on AA content differences. Additionally, we explored the predictive capabilities of multiple genomic selection (GS) methods for selecting fish fillets with high AA contents. Our results will not only reveal the key processes governing fillet AA content variations but also aid in formulating breeding strategies to enhance the fillet nutrient quality of common carp.

## 2. Materials and Methods

### 2.1. Fish Collection

This study was approved by the Animal Care and Use Committee of the Chinese Academy of Fishery Sciences under the established recommendations for the care and use of animals. Previously, 304 one-year-old common carp were raised together in a single pond for one year and fed with the same feed (Tongwei, China). The feed is composed of approximately 30% protein, 15% ash, 12.5% moisture, and 5% lipid. The muscle sample below the dorsal fin of each sample was collected to quantify the AA compositions. The liver tissues of these fish were sampled for transcriptome analysis and qPCR validation. To ensure that the post-mortem time was consistent among samples, the fillet of each fish was sampled within 10 min after euthanasia using MS-222.

### 2.2. AA Quantification in Fillets

Herein, the studied AAs were the combined AAs in the fillet rather than the free AAs in the serum. The fillet AA composition was analyzed using a high-performance liquid chromatograph (1260 Infinity II, Agilent, Santa Clara, CA, USA), following the method described by Zhang et al. [20]. Muscle samples of common carp were placed in a 20 mL hydrolysis tube, and 16 mL of 6 mol/L hydrochloric acid solution was added. The mixture was hydrolyzed at 110 °C for about 22 h. After cooling, the contents were transferred to a 10 mL centrifuge tube with deionized water and diluted to 5 mL. NaOH was added to adjust the pH to 9~11 in a 5 mL plastic centrifuge tube. Then, 250 μL of 1 mol/L triethylamine acetonitrile solution was added, followed by 250 μL of 0.1 mol/L phenyl isothiocyanate acetonitrile solution. The mixture was placed at room temperature for one hour, and then 2 mL of n-hexane was added. After shaking for 10 min, the lower phase was collected and filtered through a 0.22 μm aqueous membrane. Chromatographic separation was performed using an Agilent C18 column (4.6 × 250 mm, 5 μm), and detection was carried out at a wavelength of 254 nm. The retention times of different AAs in each sample were compared to those of the 17-component standard mixture (Nu-Chek Prep, Elysian, MN, USA). The content of each AA was determined as follows:

$$X=\frac{c\times N\times V}{m\times 1000}$$

where X denotes the absolute content of AA (μg/g); *c* represents the concentration of each AA (mg/L); V stands for the constant volume (mL); N symbolizes the dilution factor; and *m* indicates the sample weight (g).

Asp and Glu are the predominant AAs that contribute to the umami taste perception [21], and the content of total umami AA is the sum of Asp and Glu. Some AAs, including Arg, Ile, Leu, Met, Phe, His, and Val, exhibit a more bitter taste than the other AAs [22]. Thr, Ala, Ser, Pro, and Gly impart a sweet taste [23], and the content of total sweet AA is the sum of these AAs. HEAAs include Thr, Val, Met, Ile, Leu, His, Phe, Lys, and Trp [24]. The correlation between any two AA contents was calculated using the “cor.test” package. The correlations were then visualized using the “ggplot2” package [25]. To understand the AA content discrepancy among samples, principal component analysis (PCA) was performed using the “Factoextra” package, and the plot was generated using the “FactoMineR” package [26]. The AAs were clustered based on their correlations with the ‘single’ clustering method and the ‘euclidean’ distance method.

### 2.3. Estimating the Heritabilities of the AA Contents

Previously, we sequenced the genomes of these 304 samples, and the data were deposited at the NCBI SRA database (Accession: PRJNA1059144). After alignment to the reference genome [27] and filtration, 2,757,424 high-quality SNPs were retained for subsequent analysis and are available at figshare (https://figshare.com/s/2946ad101df2c2870e56 accessed on 10 February 2025). For the content of each AA, heritability was estimated using HIBLUP [28] based on these high-quality SNPs with the parameters of --single-trait, --add, and --vc-method AI. Body weight was included as a covariate. To estimate variance components, $\sigma_{p}^{2}$ denotes the total variance of the predicted value, $\sigma_{u}^{2}$ is the additive genetic variance, and $\sigma_{e}^{2}$ represents the residual variance. Thus, $\sigma_{p}^{2}=\sigma_{u}^{2}+\sigma_{e}^{2}$. The heritability h^2^ is equal to the ratio of $\sigma_{u}^{2}$ to $\sigma_{p}^{2}$, and is classified as low (<0.2), medium (0.2~0.4), or high (>0.4) [29].

### 2.4. Genome-Wide Association Study

These high-quality SNPs were used for genome-wide association studies (GWASs) utilizing the mixed linear model (MLM), which was implemented in GEMMA (v 0.98.1) [30]. The centered kinship matrix was generated using GEMMA and added to the GWASs as a covariate. The Wald test was employed to determine the significance thresholds. The suggestive threshold and significance threshold were set by dividing 100 and 10 by the total number of SNPs, respectively. Following Shim et al. Method [31], we estimated the phenotypic variance explained (PVE) of each associated SNP. Manhattan plots and quantile–quantile (Q-Q) plots were constructed with the “rMVP” package [32]. The functions of all associated SNPs to the closest genes were predicted with Annovar [33].

If one gene was distributed in 300 kb upstream and downstream of one associated SNP with each trait, identified with bedtools (v 2.30.0) [34], this gene was considered to be a candidate associated with this trait. To explore biological processes preferred by the candidate genes, we performed GO functional enrichments with TBtools-II (v 1.6) [35]. All *p* values were adjusted using the Benjamini–Hochberg (BH) method. A GO term with an adjusted *p* value < 0.05 was considered to be significant.

### 2.5. Transcriptomic Analysis to Identify DEGs Related to AA Contents

Total RNA from the liver tissue of each fish from these 304 samples was extracted using TRIzol reagent (Qiagen, San Diego, CA, USA) according to the manufacturer’s protocol. If the RNA integrity number (RIN) was larger than 7, as detected using the LabChip GX Touch (Perkin Elmer, Waltham, MA, USA), and the integrity of RNA was checked using agarose gel electrophoresis, the RNA of this sample was retained to construct the sequencing library with Stranded RNA Library Prep Kit (Illumina, San Diego, CA, USA). The library was sequenced on an Illumina Novaseq 6000 platform (Illumina, USA) with the 150 bp pair-end mode.

The raw reads were cleaned using fastp (v 3.3) [36] and then aligned to the common carp reference genome [37] using HISAT2 (v 2.0.5) [38]. Fragments per kilobase of exon model per million (FPKM) was calculated to represent the expression level of each gene, and read counts were obtained using FeatureCounts [39]. To identify the DEGs related to each AA concentration, the transcriptome data of 30 samples with the highest contents, set as the experimental group, were compared with those of the 30 samples with the lowest contents. DESeq2 [40] was employed to identify DEGs with adjusted *p* value < 0.05 and fold change > 2. A volcano plot was generated using the ggplot2 package to visualize the DEG expressions. With the FPKM values of DEGs, the PCA analysis was performed to classify samples from two groups with the ‘Factoextra’ package [26]. GO analyses were performed using the TBtools-II [35] to elucidate the preferred functions of the DEGs. All *p* values were adjusted using the BH method. A GO term with an adjusted value < 0.05 was considered to be significant.

### 2.6. Core Genes of the THEAA Concentration and PPI Network Construction

To identify the core genes detected by both GWAS and DEG, for each AA, we compared the associated genes annotated by GWAS with DEGs. If one gene was in proximity to the annotated with GWAS and had differential expression between the low-content and high-content group, this gene was considered a core gene related to the content of this AA. The differences in expression might be attributed to genetic variations.

For the THEAA concentration, the core genes were mapped to the search tool for retrieval of interacting genes (STRING) [41] to generate a protein–protein interaction (PPI) network with a minimum required interaction score of 0.15, network type as a full STRING network, and meaning of network edges as evidence. Then, functional modules of the network were explored using the ‘Analyze Network’ plugin in Cytoscape (v 3.10.3) [42]. The genes in the network were classified into three types: hub genes, secondary-connected genes, and peripheral genes.

### 2.7. qRT-PCR Validation of the Core Genes Related to THEAA Content

From the samples of which transcriptomes were sequenced, five fish with extremely high THEAA content and five fish with extremely low THEAA content comprised the high-THEAA and low-THEAA groups, respectively. To comprehensively validate the differential expression, we selected genes based on two criteria: (1) their positions in the PPI network topology, including hub genes, secondary-connected genes, peripheral genes, and non-network genes; and (2) the consistency between their functions and the preferred GO terms of DEGs. The selected hub gene was the excision repair cross-complementation group 6-like (ERCC6), and the secondary-connected gene was the type-2 angiotensin II receptor-like (AGTR2). The peripheral genes were arfgap with rhoGAP domain, ankyrin repeat and PH domain 1a (ARAP1A), tripartite motif 39 (TRIM39), p2x purinoceptor 3-like (P2RX3), lectin, and globoside alpha-1,3-N-acetylgalactosaminyltransferase 1-like (GBGT1). The slam family member 7-like (SLAMF7) and dynein regulatory complex protein 10-like (DRC10) were non-network genes. The expression patterns of these nine selected core genes in the high-THEAA and low-THEAA groups were validated using quantitative real-time PCR (qRT-qPCR).

Total RNA was extracted from liver tissue and reverse transcribed into cDNA using the HiScript II Reverse Transcriptase kit (Vazyme, Nanjing, China). Primers for the genes were designed with Primer 6 (Table S1). The β-actin gene was used as the endogenous control. The qPCR reactions were set up using SYBR qPCR master mix (Vazyme, China) and operated on the QuantReady K9600 system (QuantGene, Shanghai, China). The 2-ΔΔCT method was employed to analyze their expression levels. For each gene, the expression difference between the two groups was compared with the Wilcoxon rank-sum test (one-tailed). If the *p* value was less than 0.05, the gene was confirmed to be differentially expressed.

### 2.8. Genome Selection Analysis

For each AA content, together with the associated SNPs, twelve genome selection (GS) models in the BWGS [43] package were used to predict breeding values (BVs). For each model, ten independent cross-validation replicates were carried out. In each individual replicate, we randomly selected a training set and a validation set from the 304 accessions, adopting a size ratio of 9:1 for the two sets. The associated SNPs and the AA contents within the reference group served as the training data for each method to generate the GS model. Subsequently, the genotypes of the samples in the validation set were input into the trained model to predict the BVs. To assess the predictive performance of the GS method, for each replicate, the Pearson correlation coefficient was computed between the actual contents and the predicted BVs of the samples in the validation set. The mean correlation coefficient, along with its corresponding standard deviation (SD), was then calculated across all ten replicates for each GS method. The mean coefficient served as an indicator of the predictive ability of each GS method.

## 3. Results

### 3.1. Heterogeneities in the Levels of Fillet AAs

**Figure 1:**
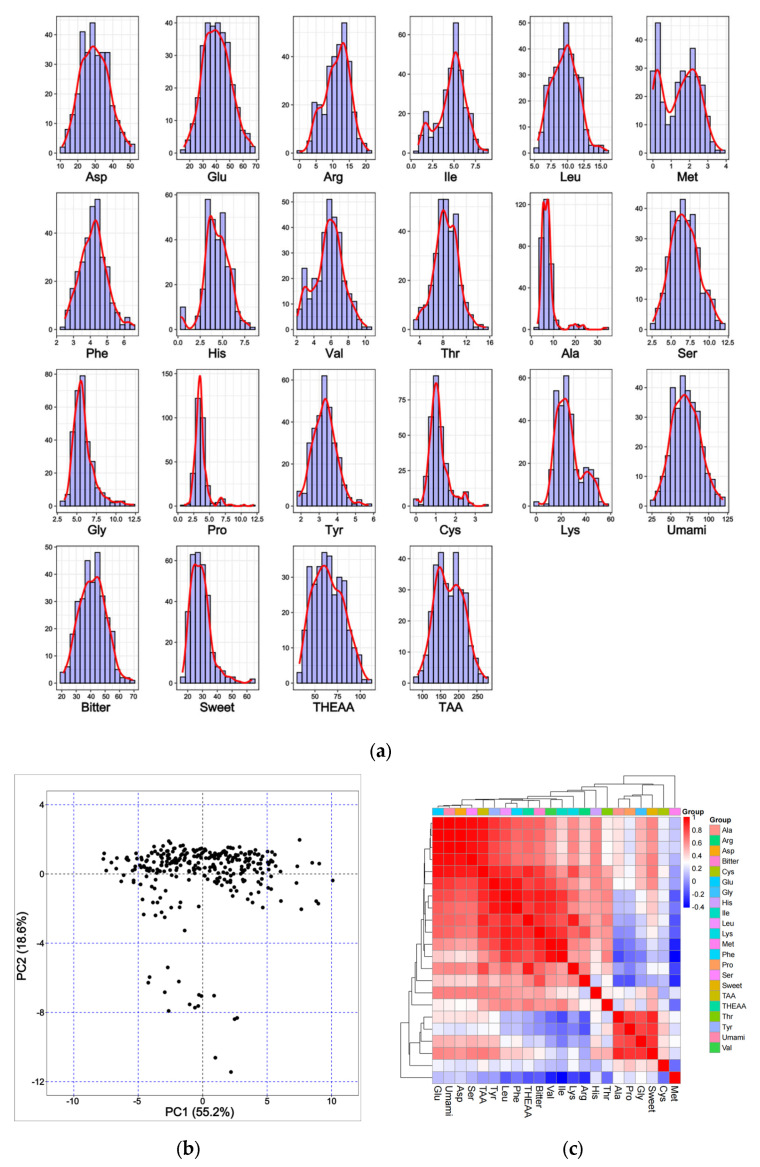
Summary of 22 AA content distributions. (**a**) The content distributions of 22 AAs. (**b**) PCA clustering common carp samples using the contents of 22 AAs. (**c**) A heatmap of the pairwise Pearson’s correlation among 22 AA contents. These 22 AAs were clustered into three main groups.

In the fillets of common carp, we identified 17 single AAs, excluding Trp, Asn, and Gln (Table S2). During the acid hydrolysis, Trp is destroyed while Gln and Asn are deamidated to Glu and Asp, respectively [44]. In general, the contents of 17 single AAs and 5 AA groups (umami AA, bitter AA, sweet AA, HEAA, and TAA) followed the normal distributions (Figure 1a). The concentration of total HEAA (THEAA) reached 64.76 mg/g, consisting of 37.14% of the total amino acid (TAA) content. The concentration of umami AA (70.02 mg/g) was higher than that of bitter AA (41.41 mg/g) and sweet AA (32.44 mg/g). The concentration of these AAs varied widely among the 304 accessions. The coefficient of variations (CVs) of the AAs ranged from 19% to 66%, indicating a high diversity in AA content among individuals (Table 1). The PCA clustering of all accessions based on the contents of all AAs showed that most individuals were discretely distributed (Figure 1b).

Among 231 pairs consisting of any two AAs, the coefficients of 198 pairs were positive and greater than the threshold of 0.113 at the *p* = 0.05 significance level (Figure 1c and Table S3). Another 12 pairs had significantly negative correlations, most of which were observed between Met and other AAs. We also observed 21 pairs without significant correlations. The coefficients of 19 AA pairs were greater than 0.9, suggesting that their metabolism might be regulated by the shared processes. The THEAA concentration was positively correlated with those of most AAs but negatively correlated with that of Met. Based on the correlation coefficients, 17 single AAs and 5 AA groups could be divided into three groups. Met formed a separate group, the content of which exhibited low correlations ranging from −0.315 and 0.287 with mean coefficients of 0.005. The second group consisted of four single AAs (including Cys, Gly, Ala, and Pro) and sweet AA. Their pairwise correlation coefficients were greater than 0.374. The third cluster consisted of the other AAs, with the intra-group coefficients ranging from 0.158 to 0.997. In the second and third groups, the correlation coefficients among intra-group members exhibited a high level (0.67 and 0.69), whereas the inter-group coefficients demonstrated a relatively low magnitude.

### 3.2. High or Medium Heritabilities of AA Contents

Since all samples were fed in the same pond, we did not estimate the permanent environmental variance. The heritabilities of 17 single AAs and 5 AA groups varied substantially. The heritability of Pro was the lowest (0.169). Five single AAs and sweet AA had medium heritabilities, ranging from 0.201 to 0.329. The heritabilities of the other 11 single AAs, umami AA, bitter AA, THEAA, and TAA, were larger than 0.4 (Table S4). Notably, the estimated heritability of THEAA was 0.774, respectively. These findings indicate that the concentration diversities of most fillet AAs were mainly regulated by the genetic variances. It is possible to improve the common carp fillet AA contents through genetic improvement.

### 3.3. Genetic Variants and Candidate Genes Associated with the AA Contents

The number of associated SNPs to each AA content under the significant threshold (3.6 × 10^−6^) was much lower than the number under the suggestive threshold (3.6 × 10^−5^, Table S5). Therefore, we employed the suggestive threshold to identify the associated SNPs and candidate genes with the contents of 17 single AAs and 5 AA groups.

**Figure 2:**
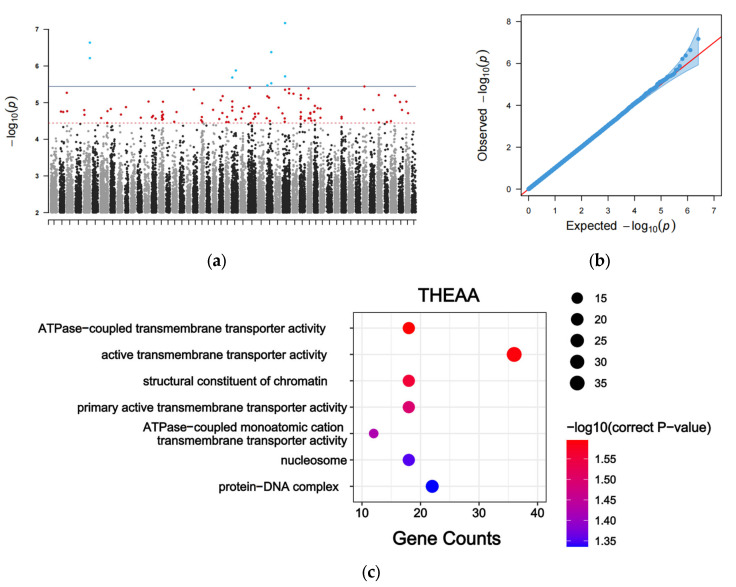
GWAS of THEAA content. (**a**) Manhattan plot for GWAS in the THEAA content. The blue points represent SNPs for which *p* values are lower than the significance threshold. The red points are SNPs for which *p* values are lower than the suggestive threshold. The black and grey points represent SNPs for which *p* values are greater than these two threshold. (**b**) Q-Q plot of the THEAA content. (**c**) GO enrichment analysis of the candidate genes surrounding the associated SNPs.

A GWAS based on the THEAA concentration identified 121 significant associated SNPs (Figure 2a), most of which were distributed in the intergenic regions (Table S6). They accounted for 5.48–9.16% of the PVE. These results indicated that the THEAA concentration might be controlled by multiple genetic loci. The Q-Q plot indicated that the statistical model used for GWAS was reasonable and reliable (Figure 2b). Among these SNPs, 3 and 62 were located in the exonic and intronic regions of protein-coding genes, respectively. We scanned the 300 kb region upstream and downstream of the 121 SNPs and identified 1998 candidate genes linked to the THEAA content. They preferred transmembrane transporter activity and protein–DNA complex (Figure 2c).

**Figure 3:**
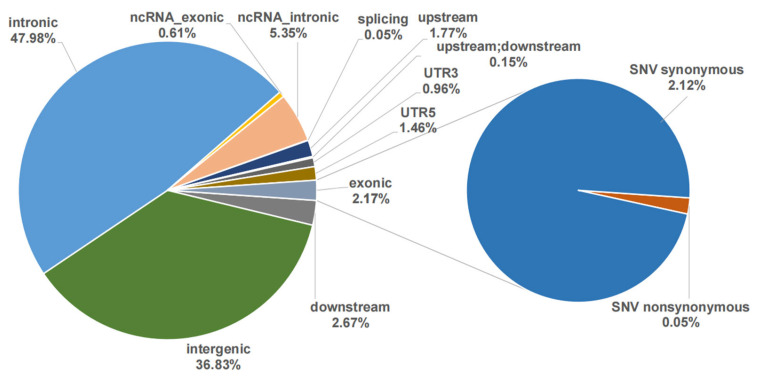
Functional annotation of the associated SNPs to their closest genes.

We also identified SNPs associated with the contents of 17 single AAs and the other 4 AA groups (Figures S1 and S2). The number of significant SNPs varied among different AAs, ranging from 46 for Glu to 330 for Pro (Table S5). In total, 1974 SNPs were associated with at least one AA content, where 872 SNPs were within the A subgenome and 933 within the B subgenome. These SNPs were close to 1769 genes (Table S6). The chromosomes A7 (107) and B22 (87) had the most SNPs associated with the AA metabolism. Nearly 89.36% of SNPs were located in intergenic or intronic regions, whereas only 2.18% were located in exonic regions (Figure 3). The proportion of synonymous SNPs (2.13%) was higher than that of non-synonymous SNPs (0.05%). Among these SNPs, 499 SNPs were associated with at least two AA traits (Table S6). These SNPs might regulate multiple AA metabolic processes. The SNPs of NC_056573.1:8354628 and NW_024879219.1:379410 exhibit the highest degree of association with amino acids, with a total of 10 distinct amino acids being involved.

We detected the candidate genes associated with different AA concentrations and their preferred GO terms (Tables S6 and S7). In total, 9046 candidate genes, including 7019 protein-coding genes, 961 ncRNAs, and 874 pseudogenes, were possibly related to the concentrations of at least two AAs (Table S8). Among the candidate genes, three protein-coding genes along with four non-coding RNAs (ncRNAs) were associated with the largest number of traits (14). A total of 88 GO terms were enriched in at least two AAs (Table S9). The single AAs of the same type might share the same biological processes. The olfactory receptor activity was enriched in the metabolic processes of Leu, Met, Phe, Tyr, Val, and bitter AA, where Leu, Met, Phe, and Val belong to bitter AA. Likewise, the umami AA, Asp, and Glu shared 14 common processes. AAs of different types also preferred the same processes. Although Asp and Met belong to the umami AA and bitter AA, respectively, the G protein-coupled dopamine receptor signaling pathway was observed to be involved in the metabolism of these two AAs (Table S9).

### 3.4. DEGs Related to the AA Concentrations

Among 304 samples, the RNA qualities of 172 individuals were in full compliance with the stringent criteria for RNA-seq library construction. The average clean transcriptome data per sample was 6.2 Gb (Table S10). Of these, 85.19% of reads were successfully mapped to the genome. For each single AA and each AA group, both the high-content group and the low-content group consisted of 30 biological replicates to ensure data reliability.

**Figure 4:**
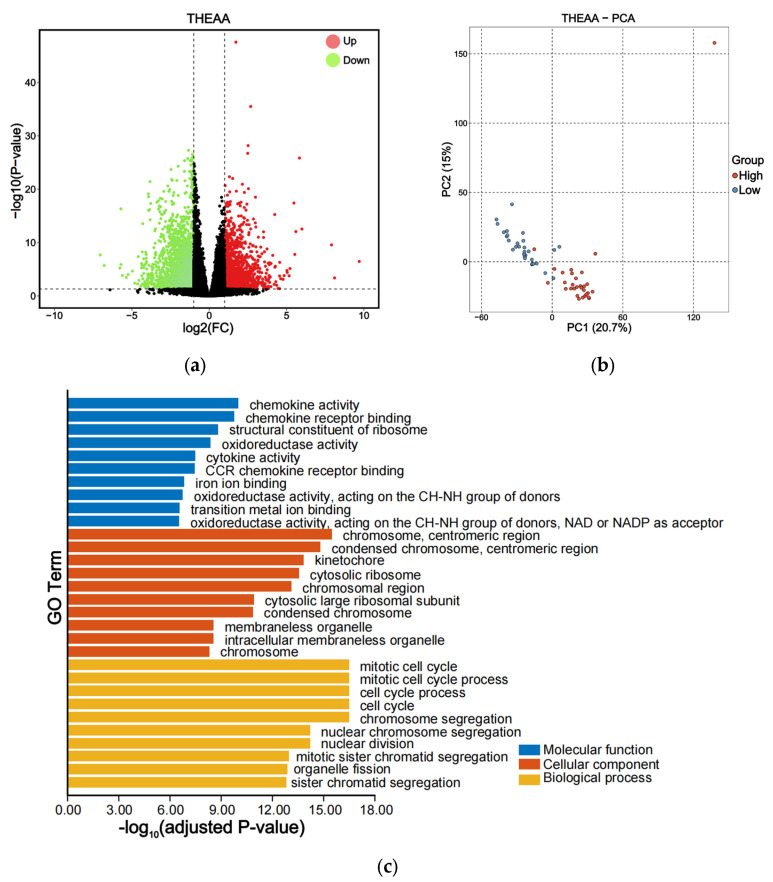
Transcriptional expression patterns in the THEAA contents. (**a**) Volcano plot of DEGs. Red and green points indicate significantly up-regulated and down-regulated genes, the black points indicate genes without differential expression, respectively. (**b**) PCA analysis to differentiate the low-content group from the high-content group. (**c**) GO enrichment analysis of DEGs.

The average THEAA concentrations in the low group and high group were 37.36 and 88.33 mg/g, respectively. In the pairwise comparison between two groups, 4727 DEGs were identified, including 1793 up-regulated genes and 2934 down-regulated genes (Figure 4a and Table S11). The PCA analysis of 60 libraries showed that the inter-group samples were separated while the intra-group samples were clustered together, indicating that these two groups exhibited distinct gene expression patterns (Figure 4b). The identified DEGs were further analyzed through GO enrichment (Figure 4c and Table S12). Within the biological process category, the predominant GO terms included mitotic cell cycle, chromosome segregation, nuclear division, and organelle fission. For the cellular component category, the leading GO terms comprised ribosome, membraneless organelle, and protein–DNA complex. Yeast grows fast in nutrient-rich media due to proteome reallocation from AA biosynthesis to ribosome [45]. In the molecular function category, the top GO terms were cytokine activity, chemokine activity, structural constituent of ribosome, oxidoreductase activity, and ion binding.

**Figure 5:**
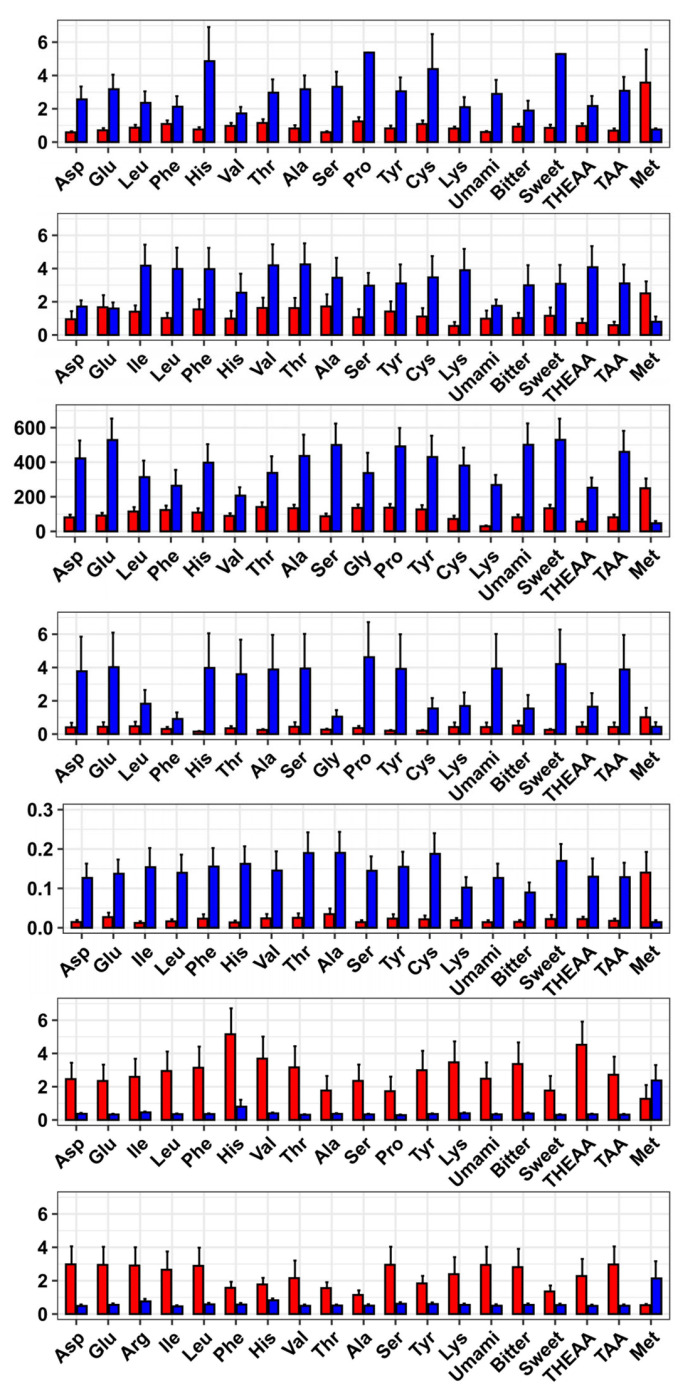
Differential expressions of seven genes in the comparisons for 19 AA contents. The genes from top to bottom are α/β-Gli-A-IV, ABI3BP, FABP10a, CSAD, B3GAT1, ssTNNT, and MCT5. The red bars display the expression levels in the high-content groups, and the blue bars show the levels in the low-content groups.

We also identified DEGs linked to 17 single AAs and the other 4 AA groups. The DEGs related to different AA concentrations and their enriched GO terms were diverse (Tables S11 and S12). With the DEGs, PCA analysis showed that the inter-group samples were clearly separated while the intra-group samples were clustered together (Figure S3). In total, 7089 candidate genes were related to the concentrations of at least two AAs (Table S13), including 6293 protein-coding genes and 694 ncRNAs. Five genes, including alpha/beta-gliadin A-IV-like (α/β-Gli-A-IV), target of Nesh-SH3-like (ABI3BP), fatty acid-binding protein 10-A, liver basic (FABP10a), cysteine sulfinic acid decarboxylase (CSAD), and galactosylgalactosylxylosylprotein 3-beta-glucuronosyltransferase 1 (B3GAT1) were up-regulated in the groups with high levels of 18 AAs but down-regulated in the high-Met-content group. Troponin T, slow skeletal muscle-like (ssTNNT), and monocarboxylate transporter 5-like (MCT5) exhibited opposite expression patterns, which were down-regulated in the groups with high levels of 18 AAs but down-regulated in the high-Met-content group (Figure 5). A total of 670 GO terms were enriched in at least two traits (Table S14), of which small molecule metabolic process, extracellular region, and iron ion binding were the top three GO terms. They were enriched in the contents of 19, 18, and 18 AAs, respectively.

### 3.5. Core Genes to Different AA Concentrations by Integrating GWAS and RNA-Seq Analysis

**Figure 6:**
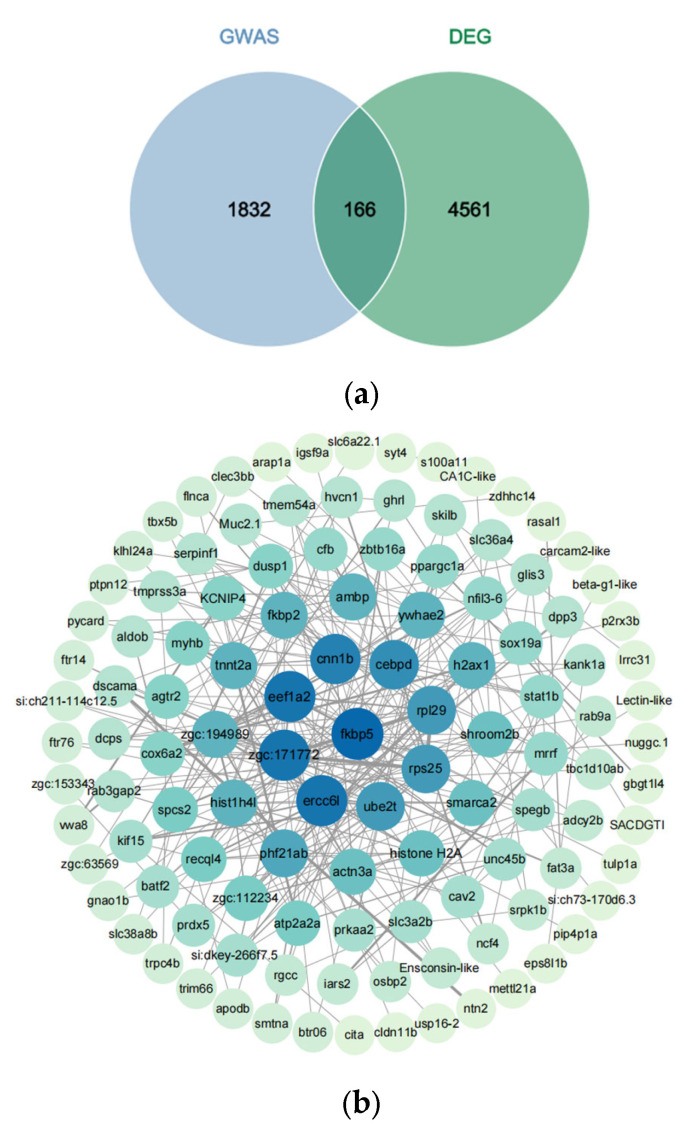
Core genes related to THEAA content. (**a**) Venn plots of core genes identified by GWAS and DEGs. (**b**) PPI network of these core genes.

To identify core genes associated with high THEAA, we compared associated genes identified by GWAS with DEGs. The characteristics of these core genes are that they are not only close to the trait-associated loci, but also show significant changes in expression between the two groups with significant content differences. For the THEAA content, 166 core genes, consisting of 151 protein-coding genes, 14 ncRNAs, and one V-segment, were not only differentially expressed between the low-THEAA group and the high-THEAA group but also associated with the THEAA content by GWAS (Figure 6a and Table S15). They were mainly predicted from the significant SNPs of the peaks on chromosomes B5, B7, and B10. Among them, 106 and 60 genes were down-regulated and up-regulated in the high-THEAA group.

We performed PPI analysis on the 166 genes and constructed a PPI network (Figure 6b). The network integrated 115 genes and there existed 9 hub genes, including FKBP prolyl isomerase 5 (FKBP5), ribosomal protein S25 (RPS25), ubiquitin conjugating enzyme E2 T (UBE2T), ERCC6, zgc:171772, eukaryotic translation elongation factor 1 alpha 2 (EEF1A2), calponin 1, basic, smooth muscle, b (CNN1B), CCAAT/enhancer binding protein delta (CEBPD), and ribosomal protein L29 (RPL29). We also detected the core genes related to the content of each AA with these two analyses (Figure S4). The core gene number was different among AAs.

### 3.6. qPCR Validated the Core Genes Related to THEAA Content

**Figure 7:**
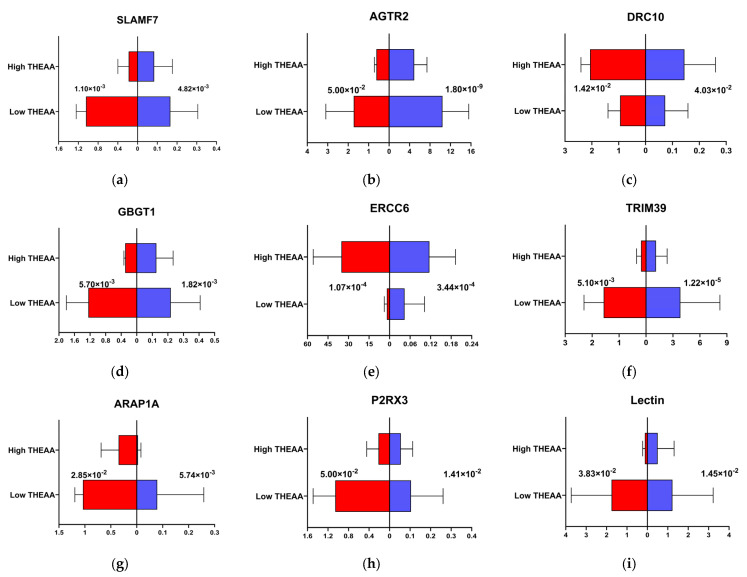
The expression trends of qRT-PCR and RNA-seq for nine core genes in the groups of low-THEAA-content and high-THEAA-content. Red is the qRT-PCR expression trends, blue is the RNA-seq expression trends. (**a**) SLAMF7; (**b**) AGTR2; (**c**) DRC10; (**d**) GBGT1; (**e**) ERCC6; (**f**) TRIM39; (**g**) ARAP1A; (**h**) P2RX3; (**i**) Lectin.

We selected nine core genes, including one hub gene, one secondary-connected gene, five peripheral genes, and two non-network genes. The qPCR results of seven core genes (SLAMF7, AGTR2, GBGT1, TRIM39, ARAP1A, P2RX3, and Lectin) were significantly down-regulated in the high-THEAA group compared with the low-THEAA group. The other two genes (DRC10 and ERCC6) were down-regulated. Their expression patterns were consistent with the results of the RNA-seq (Figure 7). The outcomes revealed that the expression patterns of these genes were closely aligned between the RT-qPCR results and the RNA-seq data. The high level of similarity confirms the reliability of the RNA-seq data, underscoring its value in identifying and analyzing gene expression related to AA metabolism.

### 3.7. Application of GS to Predict AA Concentration

**Figure 8:**
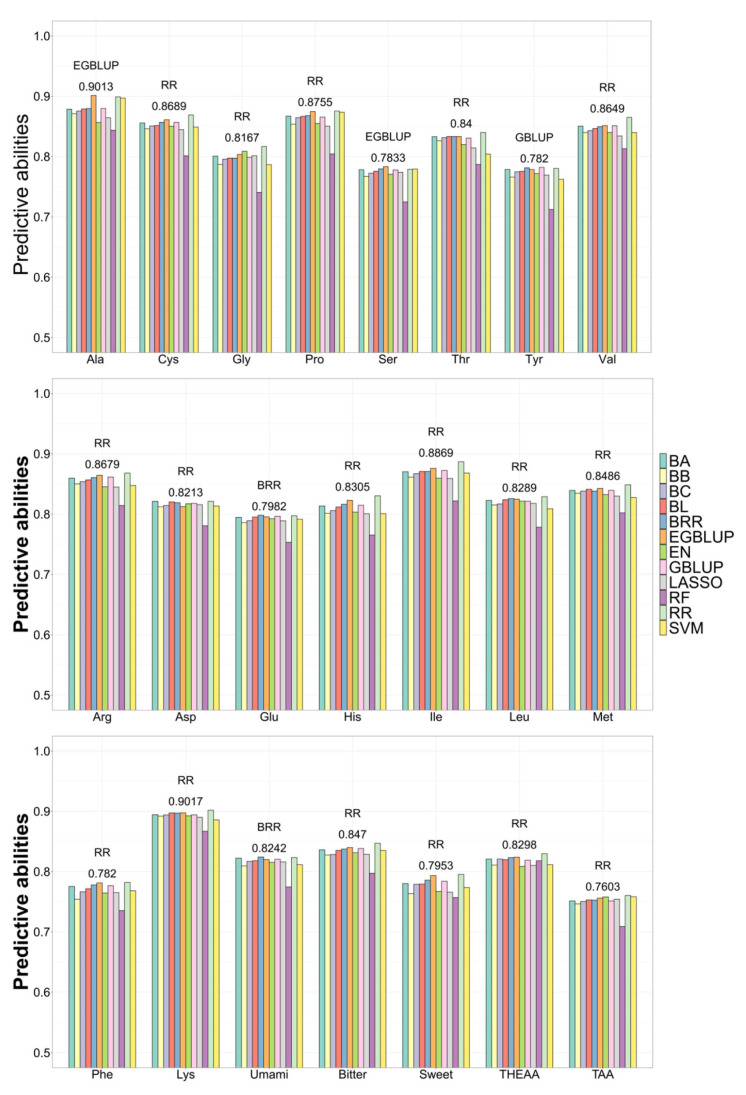
Predictive abilities of 12 GS methods for 22 AA contents.

Based on 12 GS methods, the BVs of 17 single AAs and 5 AA groups were estimated using the corresponding associated SNPs. The overall correlations for all traits between the predicted and observed values over 10 replicates ranged from 0.71 to 0.90, with the low SD less than 0.01. In each trait, 12 GS methods exhibited similar coefficients (Figure 8).

For Ala and Ser, the EGBLUP method achieved the highest correlations of 0.90 and 0.7833, respectively. For Glu and umami AA, the BRR method had the best predictive abilities of 0.7982 and 0.8242, respectively. For the other single AAs and AA groups, the RR method was superior to the other 11 methods, with the highest correlations. For HEAA-related single AAs, the highest CVs ranged from 0.78 to 0.90. These data indicated that the associated markers and the optimal GS methods were efficient at accurately predicting the fillet AA concentrations.

## 4. Discussion

The fillet contents of 22 AAs in 304 common carp varieties were investigated. The common carp fillet is rich in eight types of HEAAs, except Trp. The AA contents vary considerably among individuals. The CVs ranged from 19% to 66%, where the CV of THEAA was 25%. The differences in fillet AA contents reflect the comprehensive level difference in the AA metabolism capabilities. We performed genome and transcriptome analysis to identify associated genes and DEGs linked to each single AA and each AA group, respectively. By integrating GWAS and DEG results, we narrowed down the candidate genes to core genes. With the associated SNPs and optimal GS method, we can accurately predict the fillet AA contents of each sample. Overall, the insights gained from this research offer valuable guidance and constitute an important reference to select common carp with high AA contents, especially high THEAA content.

### 4.1. Competition and Coordination in the Metabolism of Different AAs

The AAs could be divided into three clusters. Met forms a distinct cluster from the other two clusters. Met is insignificantly or negatively correlated with the other AAs. Met is a sulfur-containing aliphatic, nonpolar α-amino acid [46]. It is the precursor of Cys [47]. Therefore, the contents of these two AAs are negatively correlated (correlation of −0.11). System y+ L transporters exhibit a unique ability to bind both cationic (Lys) and neutral AAs (Met), depending on sodium ion gradients [48]. This dual specificity suggests potential competition between Lys and Met during AA absorption, possibly leading to a negative correlation between these two AAs (correlation of −0.22).

The members within the second cluster and the third cluster were significantly and highly correlated. Many associated genes, DEGs, and their preferred processes were shared in multiple AAs, underlying the molecular basis of these high-concentration correlations. Considering the strong correlation in the content of multiple AAs, it is not necessary to conduct individual breeding for the content of a single AA, and it is feasible to increase the fillet contents of multiple AAs simultaneously.

### 4.2. Catalytic Activities of Oxidoreductases

The DEGs linked to the THEAA content prefer oxidoreductase activity. Oxidoreductases are the largest class of enzymes, comprising dehydrogenases, oxygenases, peroxidises, oxidases, and other enzymes catalyzing oxidation–reduction reactions [49]. These enzymes function in AA metabolism. Amino acid dehydrogenases, one class of oxidoreductases, transfer hydride from the Cα atom of an AA to NAD(P)+, generating α-keto acid and ammonium [50,51,52]. Aldehyde dehydrogenase 1 family member L1 (ALDH1L1), one AA dehydrogenase, performs the oxidation reaction, where an aldehyde binds to specific AAs in the catalytic domain [53]. In the NAD(P)+-binding domain, the NAD(P)+ cofactor facilitates the redox reaction catalyzed by ALDH. Down-regulation of ALDH1L1 in the high-THEAA group might decrease its oxidation activity, resulting in the elevated levels of its regulated AAs.

Pyrroline-5-carboxylate reductase (PYCR), one oxidoreductase, converts pyrroline-5-carboxylate (P5C) into Pro and is an essential enzyme in Pro synthesis [54]. Methylenetetrahydrofolate dehydrogenase 2 (MTHFD2), another oxidoreductase, and the network it regulates, participated in the import of AAs, the synthesis of Gly and Ser, and the interconversion among Asp, Met, and Cys [55]. These two genes were up-regulated in the high-THEAA group and might increase the contents of their regulated AAs. Overall, the differences in AA composition among the varieties reflect the distinctions in their corresponding oxidoreducatse activities.

### 4.3. Participation of Immune-Related Pathways to AA Metabolism

The DEGs identified in the comparison of THEAA content were enriched in cytokine activity, chemokine activity, and chemokine receptor binding. Besides IL-6, interferon γ (IFN-γ) can strongly up-regulate the mRNA expression of IDO1 [56]. TGF-β induces arginase1 (ARG1) activity, and subsequently up-regulated the IDO1 expression [57,58]. The ARG1 catabolizes L-Arginine [59]. Moreover, T lymphocyte activation induces the expression of Glu transporters and glutaminase, further increasing Glu uptake and metabolism [60]. The results demonstrated that the cytokines and chemokines regulated AA metabolism through modulating the activities of AA enzymes and transporters.

Upon activation, immune cells rewire growth, proliferation, and effector functions by acquiring energy and biomolecules, including AAs [61]. Beyond fueling increased protein synthesis, AA metabolism plays a pivotal role in supporting a wide array of immune cell functions. Activated T cells utilize Glu to energy metabolism, serve as a nitrogen source, and act as an anapleurotic substrate [62]. The AAs or AA transporters are crucial in T-cell-mediated immunity [63,64,65]. The interplay between AA metabolism and immunity occurs extensively at different levels of regulation.

Besides the oxidoreductases and immune-related pathways, we identified many novel candidate genes. There is no functional evidence yet of these genes participating in AA metabolism. Their functions should be further validated through integration with other biological techniques.

### 4.4. Application of GS and Gene-Editing Technique in Improving the Fillet AA Contents

To improve the AA content of fish fillets, animal diets are supplemented with exogenous AAs. This is a costly process for farmers. Efforts were put into replacing fish meal with other AA sources, for instance, soybean, rapeseed, and cottonseed meal [66]. However, meal production is limited, and meal is mainly supplied to livestock and poultry. Increasing the supply of exogenous AAs is not an optimal or sustainable solution to resolve the AA demands of aquaculture.

If the capacities for producing endogenous non-essential AAs and increasing the efficiency of essential AAs in fish are improved, it would not only decrease the demand for exogenous meal but also have important impacts on human health. However, research on the genetic basis and regulatory mechanisms underlying the AA composition discrepancies remains relatively scarce. The contents of most AAs in common carp fillets were of medium and high heritabilities. In soybean, the heritabilities of single AAs ranged from 0.35 to 0.71, also suggesting that genetic variants contribute to the AA contents [5]. The medium-to-high heritabilities suggested that it is feasible to utilize a genomic approach for selecting fish having high fillet AA contents. The SNPs associated with the AA contents and the optimal GS methods would be used to estimate the breeding values of the testing population and guide the selection of individuals potentially having higher fillet nutrient quality.

The GS has some limitations. It demands a representative reference population [67]. High computation and genotyping costs also pose a barrier [68]. Model accuracy is compromised by complex gene–environment interactions [69]. Compared with the GS, the CRISPR/Cas9 gene-editing system is cheap and precise, with rapid phenotypic changes [70]. Our study identified many candidate genes, which would be future editing targets.

### 4.5. Application of Other Omics Data in Identifying AA-Related Genes and Pathways

Besides genome resequencing and transcriptome, other omics data, including metabolome [71], proteomics [72], epigenomics [73], and single-cell transcriptome [74] have been widely utilized in trait-related analysis. Indeed, epigenetic regulation was demonstrated to participate in modulating AA metabolism [75]. These emerging omics datasets hold the potential to unveil intricate regulatory networks of AA metabolism, thereby offering a more comprehensive understanding and complementing our current findings.

## 5. Conclusions

We quantified the AA compositions and integrated genome and transcriptome analysis to trace the primary processes regulating the heterogeneities in the fillet AA contents. High variations in fillet AA contents among samples were observed. Most AAs showed medium-to-high heritabilities, suggesting that genetic variances mainly contributed to the AA content diversity. The GWAS was conducted to identify the SNPs and candidate genes associated with each AA content. The modulation of AA content variations by these genes might be mediated via genetic mutations. The DEGs possibly diversify the fillet AA contents through expression change. For the THEAA content, the SNPs and associated genes were involved in transporter activity, while the DEGs preferred cytokine binding, chemokine activity, oxidoreductase activity, and ion binding. The high predictive power for AA compositions of the optimal GS methods helps estimate breeding values and select individuals with putatively high AA contents. As an alternative breeding strategy, carrying out gene editing on the identified candidate genes holds the promise of rapidly obtaining new germplasms with enhanced AA content. The findings of this study not only provide new insights into the molecular mechanisms underlying the discrepancies in fish AA contents but also support developing a genomic selection strategy to improve fillet AA content in common carp.

## Figures and Tables

**Table 1:** Divergent AA profiles in common carp fillets (mg/g).

	Mean	SD	Max	Min	CV
Asp	29.73	7.98	50.82	11.01	27%
Glu	40.29	10.05	67.81	15.57	25%
Arg	11.26	3.76	20.99	0.00	33%
Ile	4.73	1.60	8.93	0.82	34%
Leu	9.64	1.97	16.06	5.51	20%
Met	1.47	0.97	3.81	0.00	66%
Phe	4.19	0.78	6.62	2.49	19%
His	4.38	1.36	8.12	0.29	31%
Val	5.74	1.66	10.18	2.06	29%
Thr	8.54	2.04	15.31	3.40	24%
Ala	7.59	3.99	34.38	2.75	53%
Ser	6.81	1.83	11.75	2.60	27%
Gly	5.86	1.39	12.29	3.38	24%
Pro	3.64	1.18	11.98	0.96	32%
Tyr	3.30	0.63	5.72	1.89	19%
Cys	1.14	0.50	3.53	0.00	44%
Lys	26.07	10.28	57.73	1.52	39%
Umami AA	70.02	17.96	118.63	26.58	26%
Bitter AA	41.41	8.98	69.25	21.06	22%
Sweet AA	32.44	7.96	75.48	18.66	25%
TAA	174.38	38.50	278.36	86.08	22%
THEAA	64.76	16.45	109.69	32.82	25%

## Data Availability

The genome sequencing data presented in the current study have been submitted to the NCBI BioProject database under the accession number PRJNA1059144. The transcriptome data presented in the current study have been submitted to the NCBI BioProject database under the accession number PRJNA1190881.

## Supplementary Materials

The following supporting information can be downloaded at: https://www.mdpi.com/article/10.3390/ani15091335/s1. Figure S1: Manhattan plots for GWAS in the content of 21 AAs; Figure S2: Quantile–quantile plots between the expected -log(P) value and the observed -log10 (P) value of 21 AA contents based on the corresponding SNPs; Figure S3: PCA analysis of the low-content and high-content groups; Figure S4: Venn plots of core genes identified by GWAS and DEGs; Table S1: qPCR validation primers of nine selected genes; Table S2: The contents of muscular AAs in common carp; Table S3: Content correlation coefficients of any two AAs; Table S4: Additive genetic variance, residual variance, and heritability of each AA content; Table S5: Associated SNPs and candidate genes for each AA content; Table S6: Functions of associated SNPs and their closest genes; Table S7: Functional enrichments of candidate genes; Table S8: Shared candidate genes by at least two AA contents; Table S9: Shared enriched GO terms by at least two AA contents; Table S10: RNA-seq alignment ratios of the low and high groups of each AA content; Table S11: Differential expression profiles related to each AA concentration; Table S12: GO enrichments of DEGs related to each AA concentration; Table S13: DEGs shared in multiple traits identified by RNA-seq; Table S14: GO terms shared in multiple traits, enriched by DEGs; Table S15: One hundred sixty-six core genes related to THEAA content covered by GWAS and DEG analysis.

## Abbreviations

The following abbreviations are used in this manuscript:

AAs	Amino acids
HEAAs	Human essential amino acids
THEAA	The concentration of total HEAA
TAA	Total amino acid
