# Supplementary material for: Genomic and Transcriptomic Profiling of Amino Acid Compositions in Common Carp Fillets
Source: Animals (Basel). 2025 May 6;15(9):1335. doi: 10.3390/ani15091335 (PMC12070895; doi:10.3390/ani15091335)
Supplement: Supplementary file 1 [file animals-15-01335-s001.zip › animals-3598969-supplementary/Supplementary Figures-v3.pdf]

Supplementary Figure S1. Manhattan Plots for GWAS in the contents of 21 AAs.

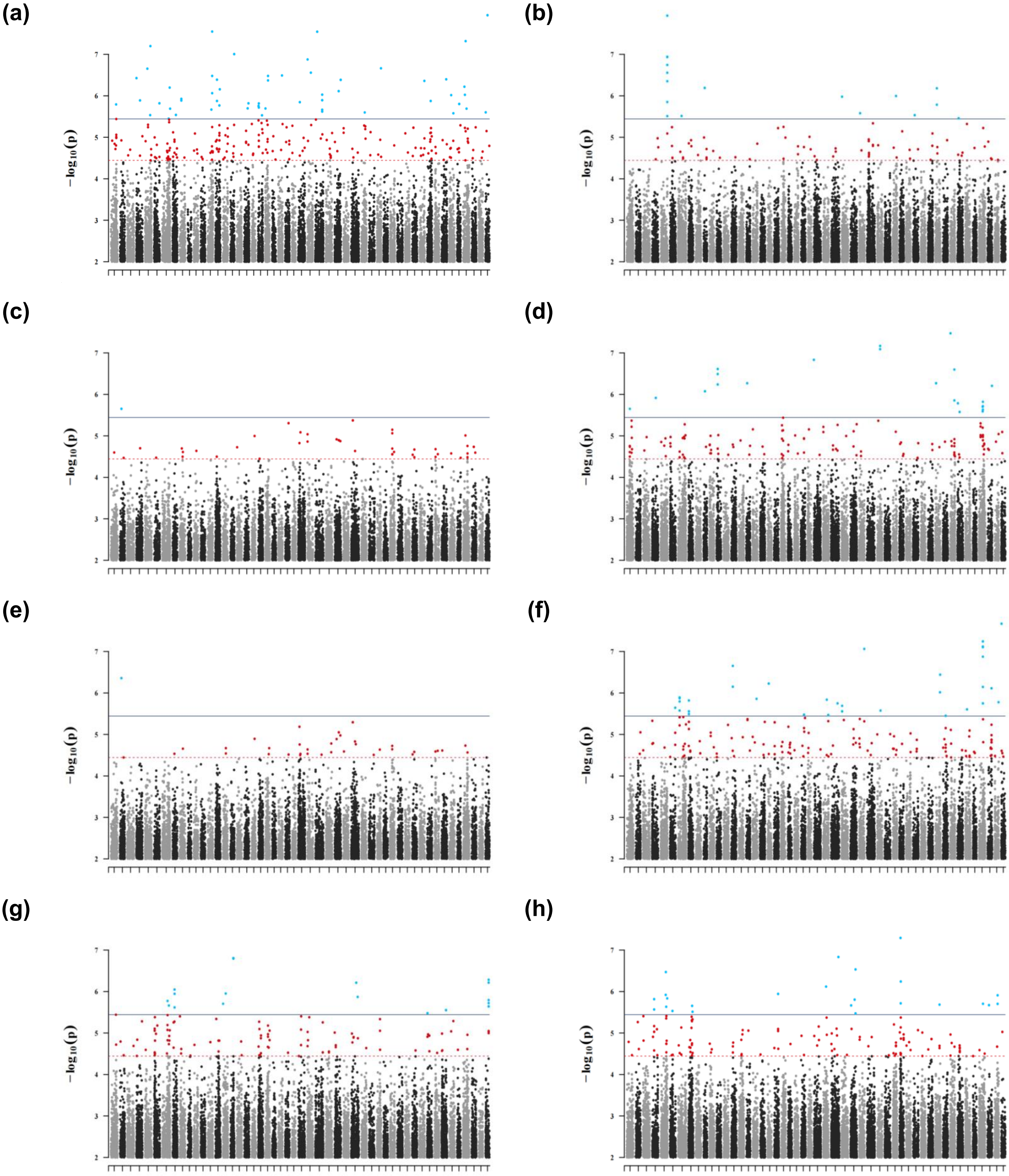

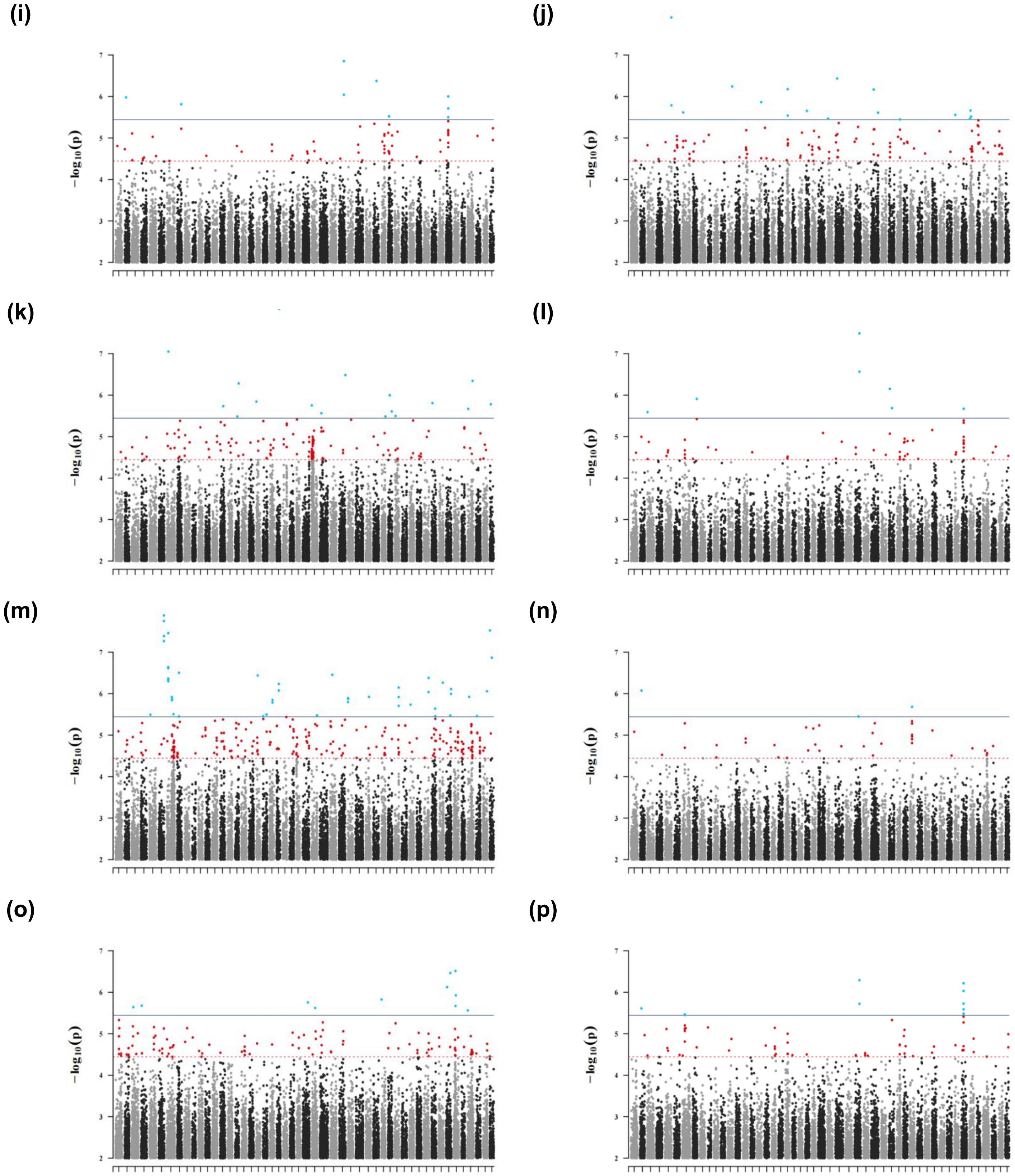
(i)
(j)
(k)
(l)
(m)
(n)
(o)
(p)
−log10(p)

(q)

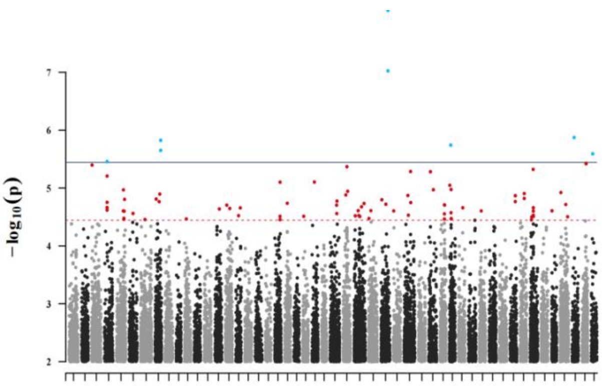

(r)

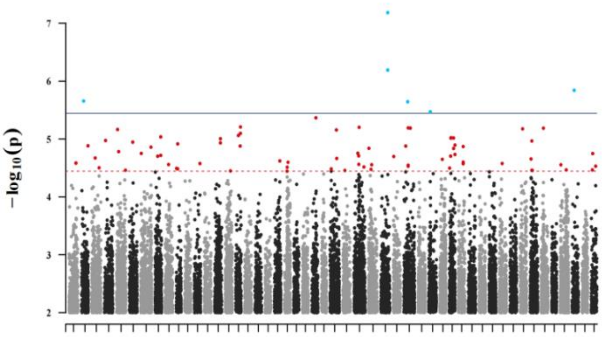

(s)

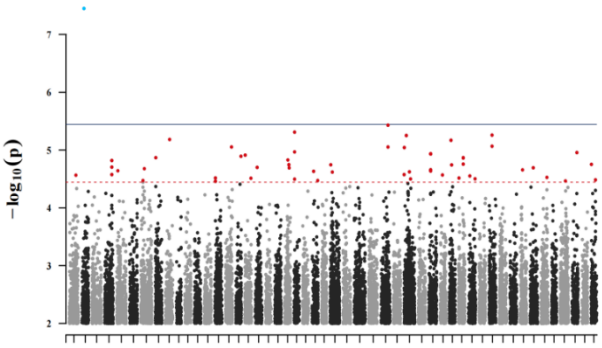

(t)

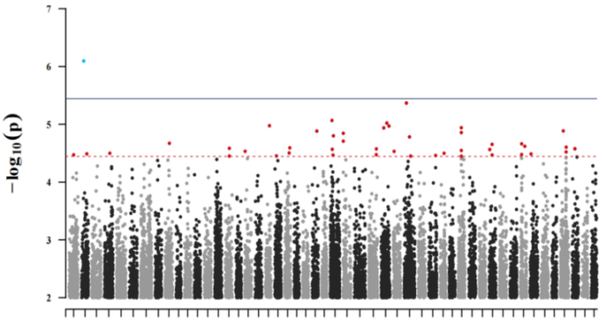

(u)

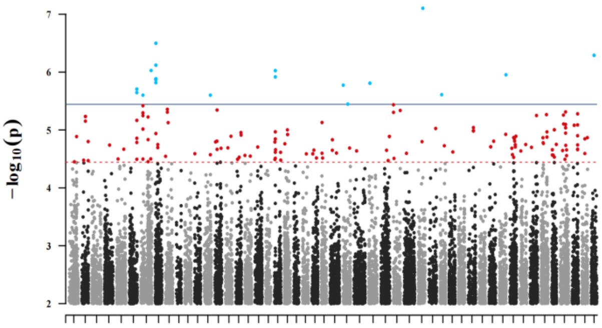

(a) :Ala; (b):Arg; (c):Asp; (d):Cys; (e):Glu; (f):Gly; (g):His; (h):Ile; (i):Leu; (j):Lys; (k):Met; (l):Phe; (m):Pro; (n):Ser; (o):Thr; (p):Tyr; (q):Val; (r):Bitter AA; (s):TAA; (t) Umami AA; (u) Sweet AA.

Supplementary Figure S2. Q-Q plots between the expected -log(P) value and the observed -log10 (P) value of 21 AA contents based on the corresponding SNPs

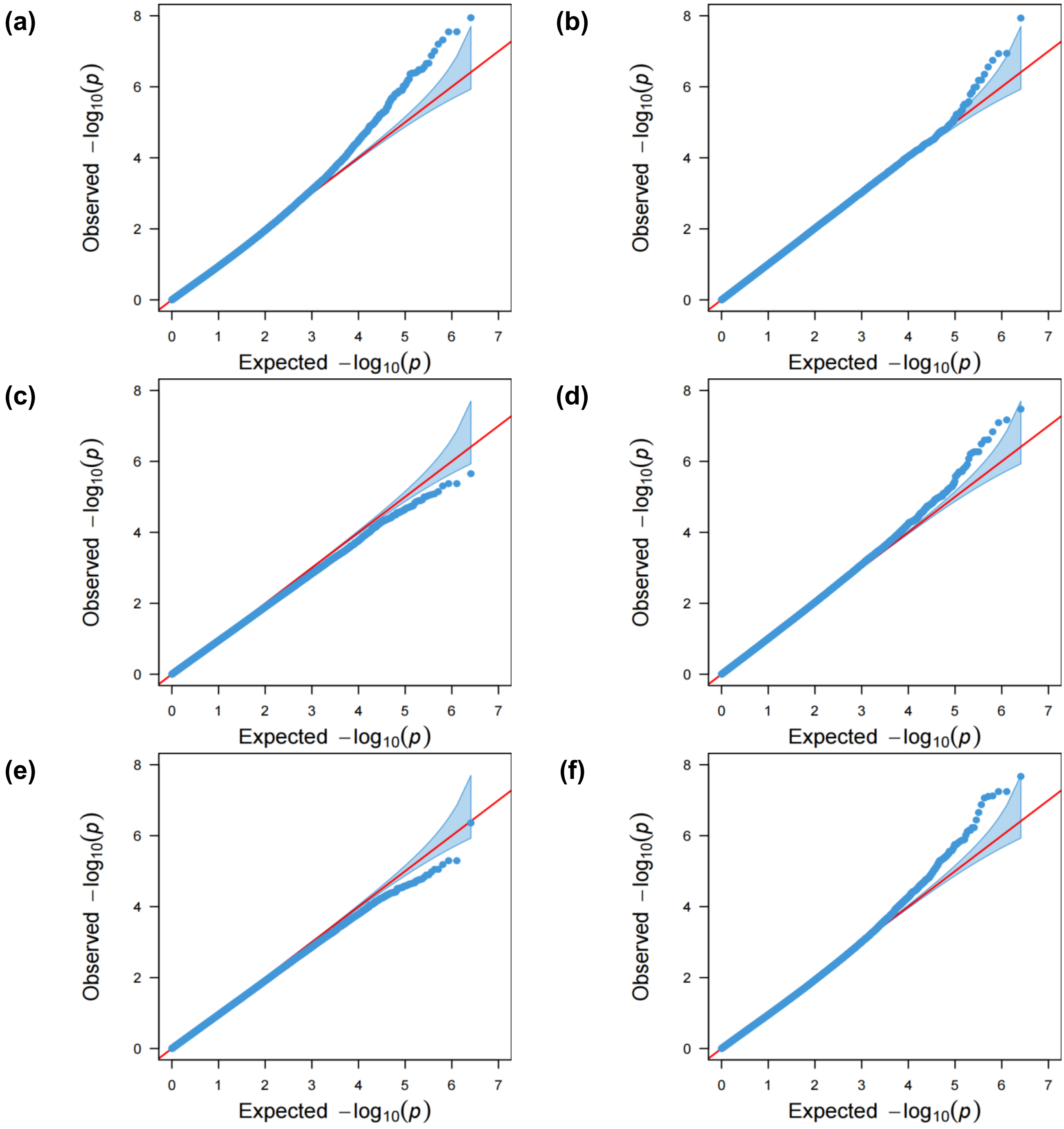

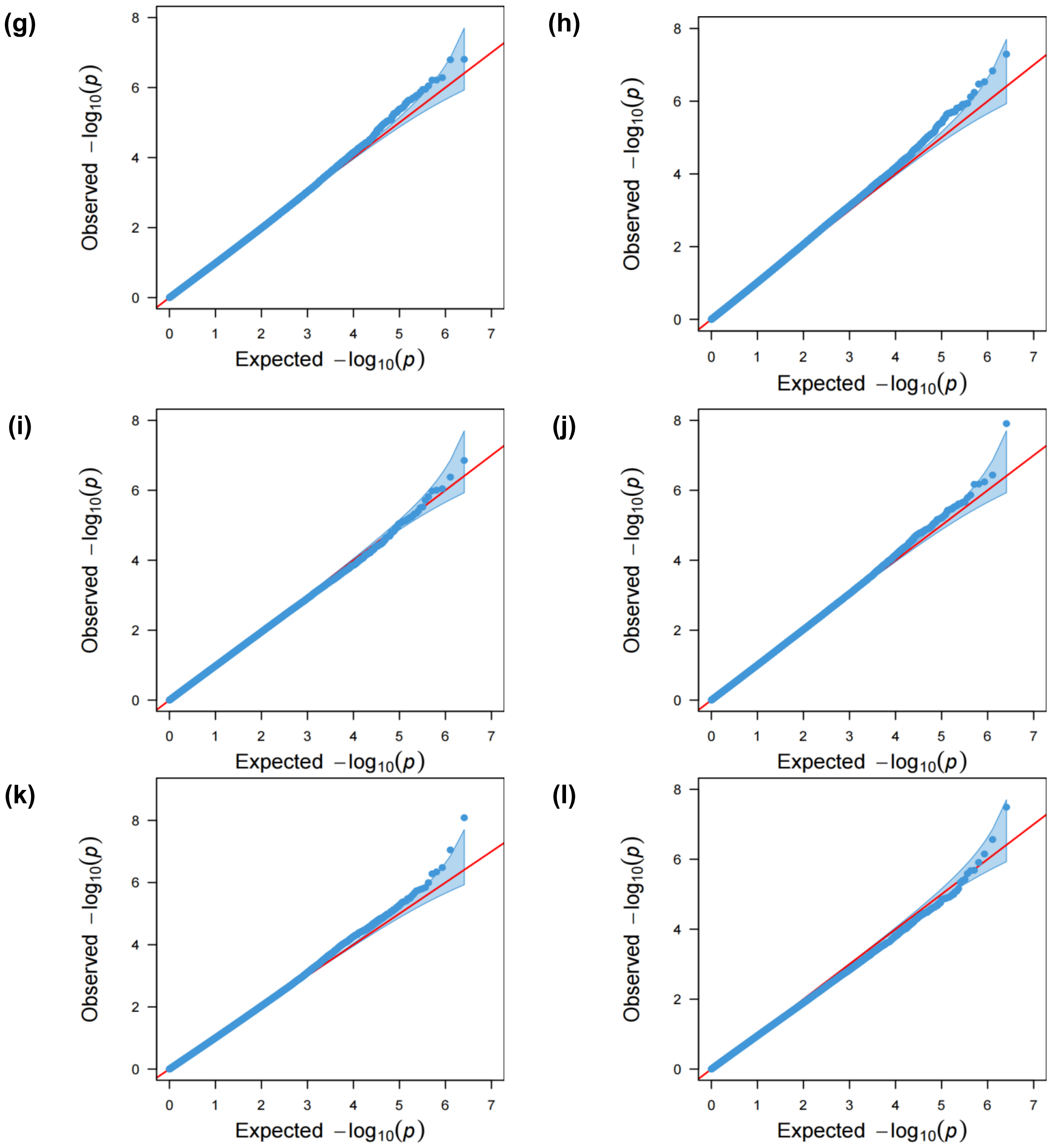
(g)
(h)
(i)
(j)
(k)
(l)
Observed $-\log_{10}(p)$
Expected $-\log_{10}(p)$
0
2
4
6
8
0 1 2 3 4 5 6 7

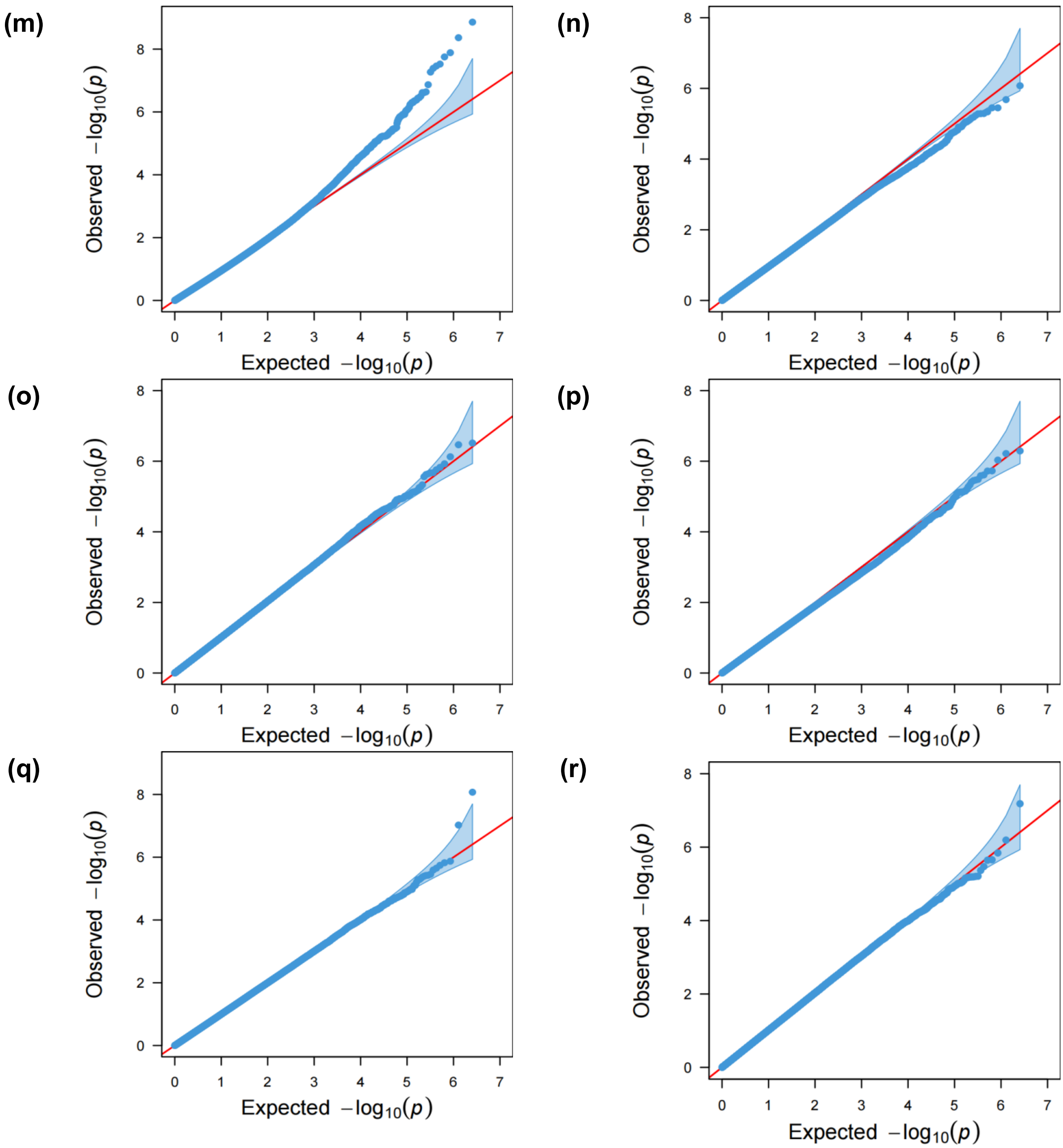

(m)
(n)
(o)
(p)
(q)
(r)
Observed $-\log_{10}(p)$
Expected $-\log_{10}(p)$

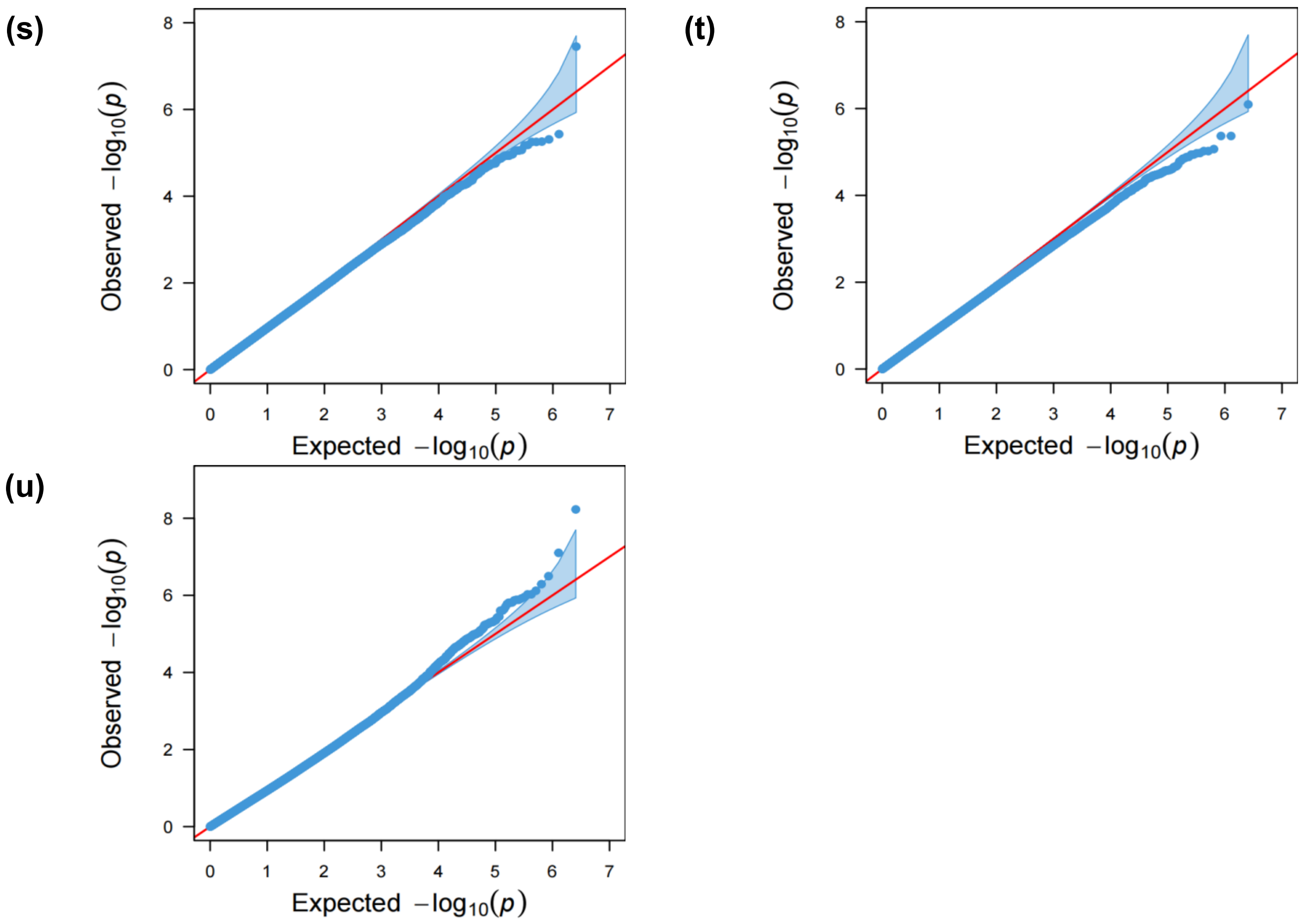

(a) :Ala; (b):Arg; (c):Asp; (d):Cys; (e):Glu; (f):Gly; (g):His; (h):Ile; (i):Leu; (j):Lys; (k):Met; (l):Phe; (m):Pro; (n):Ser; (o):Thr; (p):Tyr; (q):Val; (r):Bitter AA; (s):TAA; (t) Umami AA; (u): Sweet AA.

Supplementary Figure S3. PCA analysis of the low content and high content groups

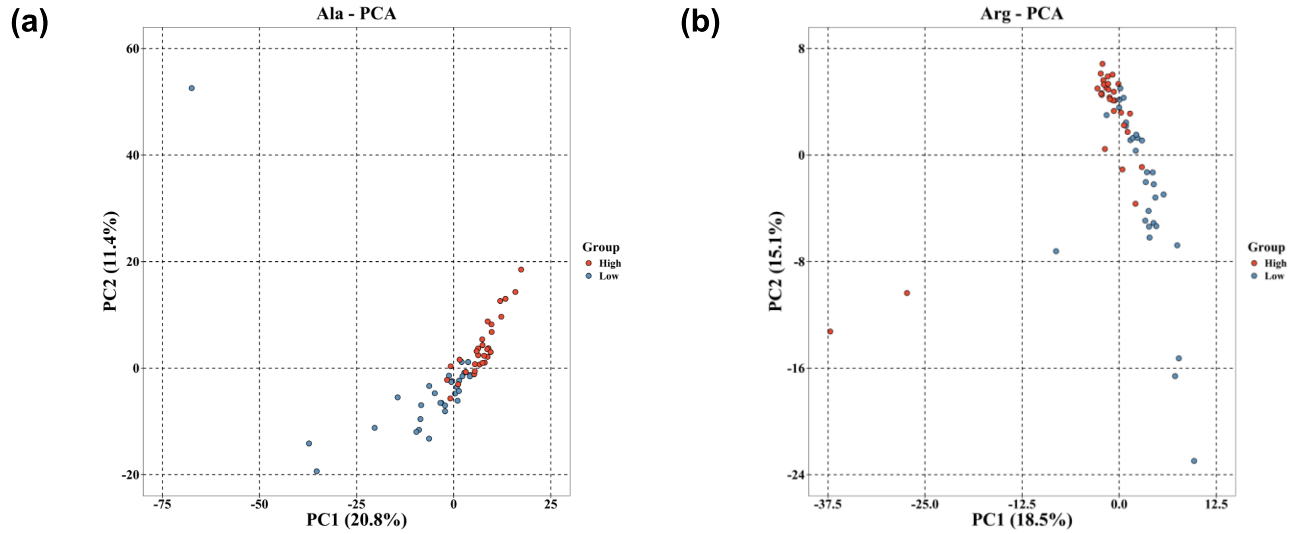

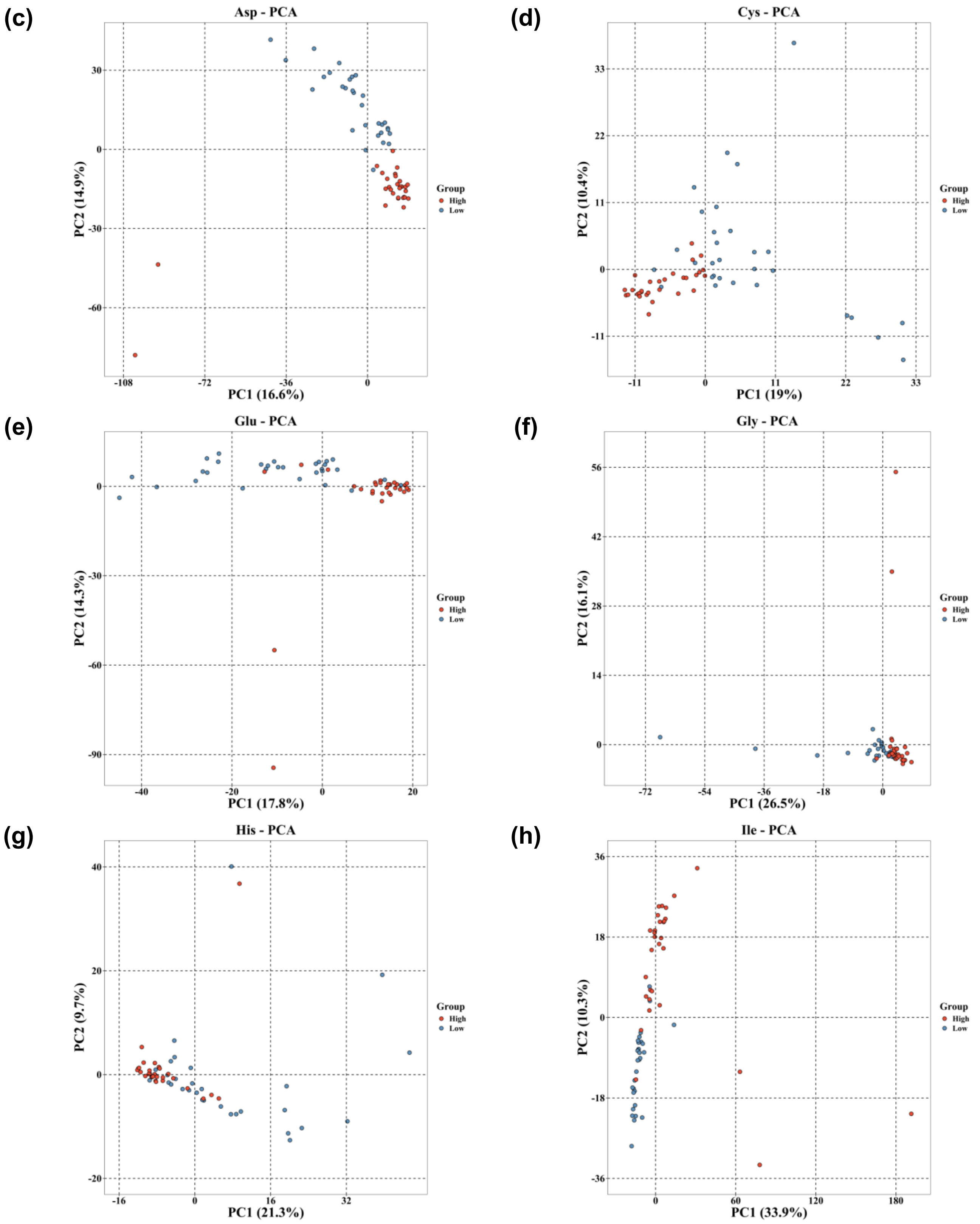

(c)
Asp - PCA
PC1 (16.6%)
PC2 (14.9%)
-108
-72
-36
0
30
0
-30
-60
Group
High
Low
(d)
Cys - PCA
PC1 (19%)
PC2 (10.4%)
-11
0
11
22
33
33
22
11
0
-11
Group
High
Low
(e)
Glu - PCA
PC1 (17.8%)
PC2 (14.3%)
-40
-20
0
20
0
-30
-60
-90
Group
High
Low
(f)
Gly - PCA
PC1 (26.5%)
PC2 (16.1%)
-72
-54
-36
-18
0
56
42
28
14
0
Group
High
Low
(g)
His - PCA
PC1 (21.3%)
PC2 (9.7%)
-16
0
16
32
40
20
0
-20
Group
High
Low
(h)
Ile - PCA
PC1 (33.9%)
PC2 (10.3%)
0
60
120
180
36
18
0
-18
-36
Group
High
Low

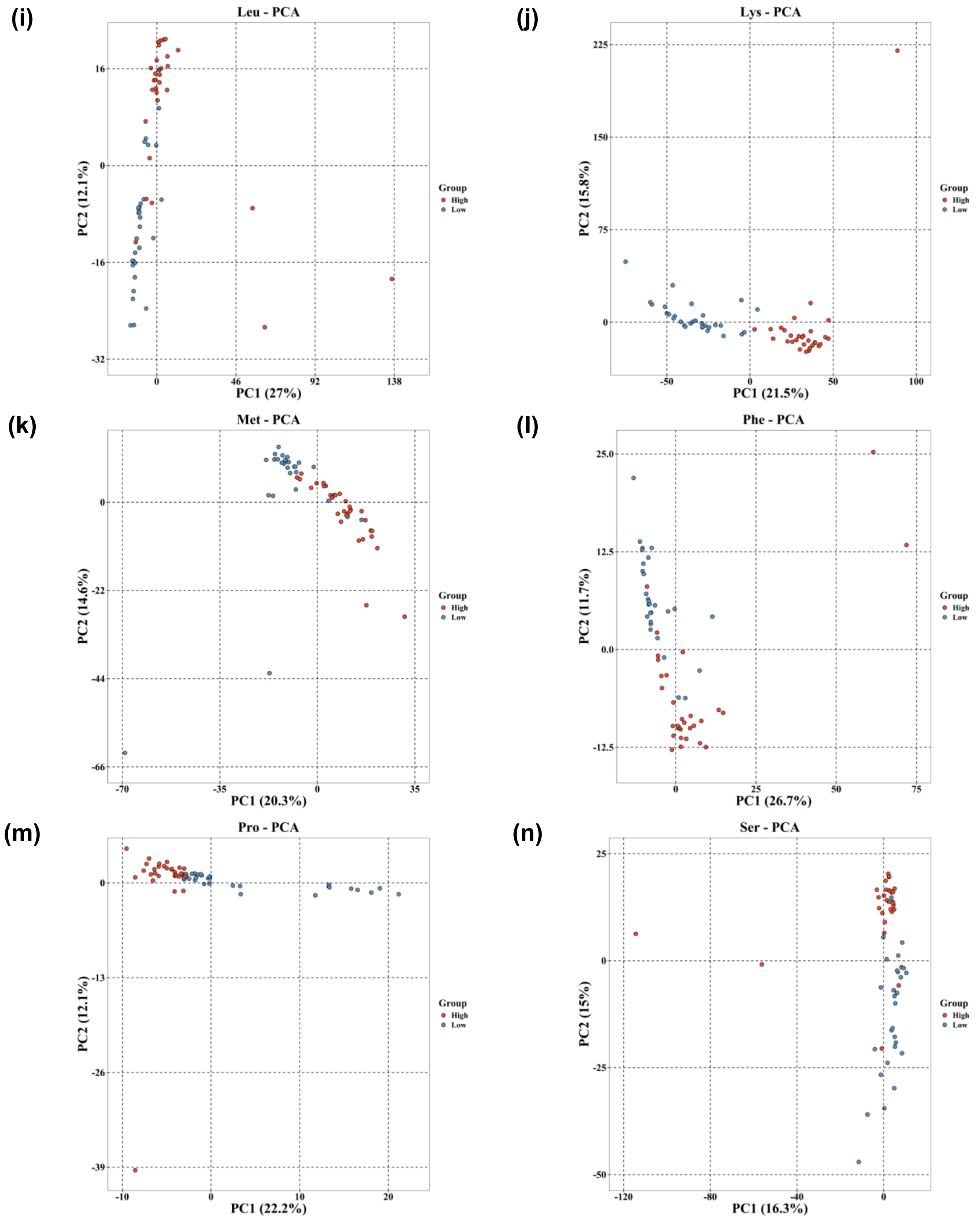

(i)
Leu - PCA
PC1 (27%)
PC2 (12.1%)
16
0
-16
-32
46
92
138
Group
High
Low
(j)
Lys - PCA
PC1 (21.5%)
PC2 (15.8%)
225
150
75
0
-50
50
100
Group
High
Low
(k)
Met - PCA
PC1 (20.3%)
PC2 (14.6%)
-22
-44
-66
-70
-35
35
Group
High
Low
(l)
Phe - PCA
PC1 (26.7%)
PC2 (11.7%)
25.0
12.5
0.0
-12.5
25
50
75
Group
High
Low
(m)
Pro - PCA
PC1 (22.2%)
PC2 (12.1%)
-13
-26
-39
-10
10
20
Group
High
Low
(n)
Ser - PCA
PC1 (16.3%)
PC2 (15%)
25
-25
-50
-120
-80
-40
Group
High
Low

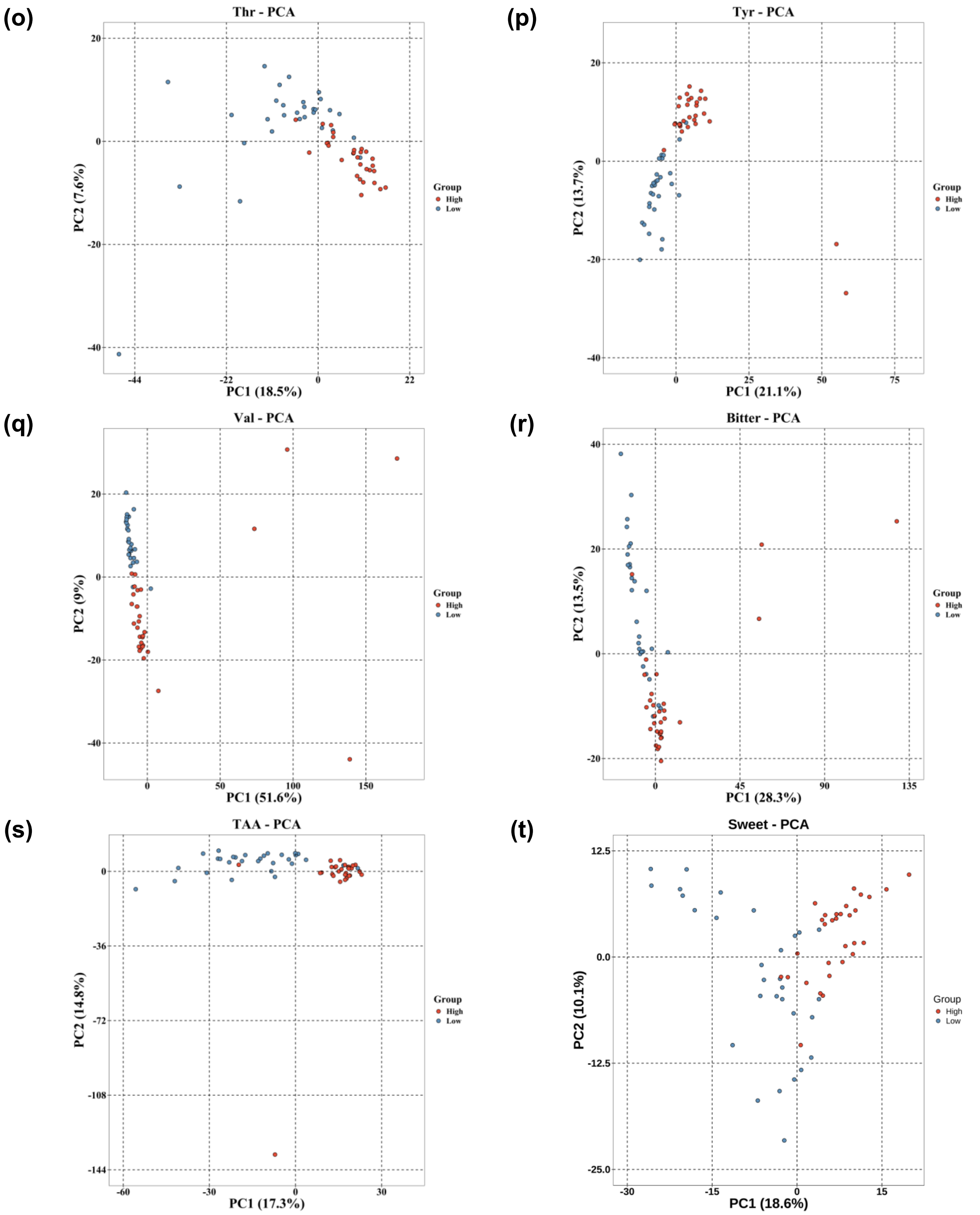

(o)
Thr - PCA
PC1 (18.5%)
PC2 (7.6%)
Group
High
Low
(p)
Tyr - PCA
PC1 (21.1%)
PC2 (13.7%)
Group
High
Low
(q)
Val - PCA
PC1 (51.6%)
PC2 (9%)
Group
High
Low
(r)
Bitter - PCA
PC1 (28.3%)
PC2 (13.5%)
Group
High
Low
(s)
TAA - PCA
PC1 (17.3%)
PC2 (14.8%)
Group
High
Low
(t)
Sweet - PCA
PC1 (18.6%)
PC2 (10.1%)
Group
High
Low

(u)

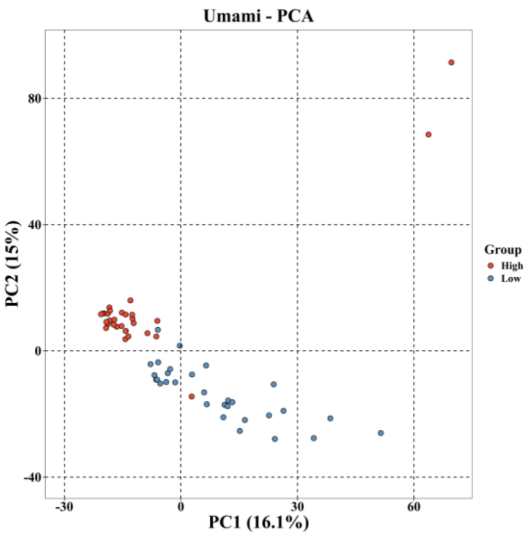

(a):Ala; (b):Arg; (c):Asp; (d):Cys; (e):Glu; (f):Gly; (g):His; (h):Ile; (i):Leu; (j):Lys; (k):Met; (l):Phe; (m):Pro; (n):Ser; (o):Thr; (p):Tyr; (q):Val; (r):Bitter AA; (s):TAA; (t):Sweet AA; (u):Umami AA.

Supplementary Figure S4. Venn plots of core genes identified by GWAS and DEGs.

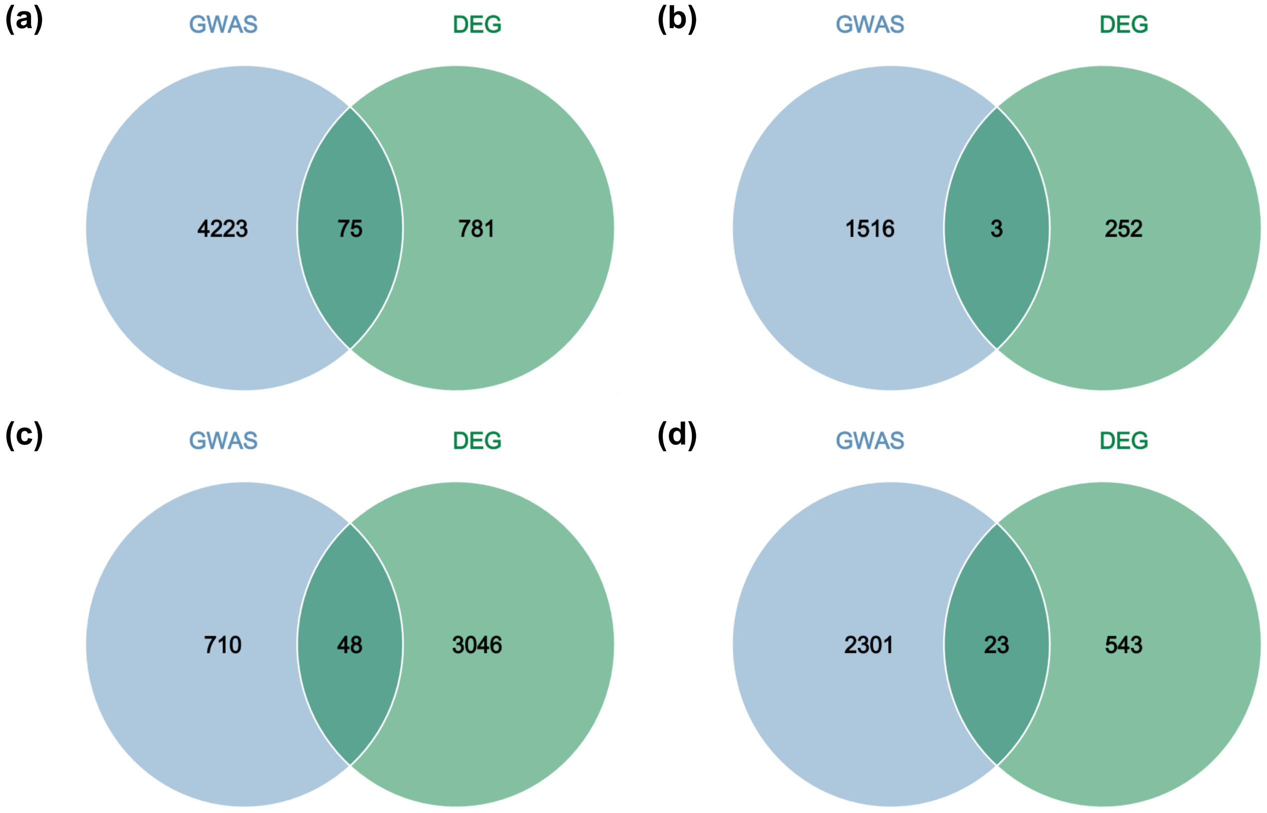

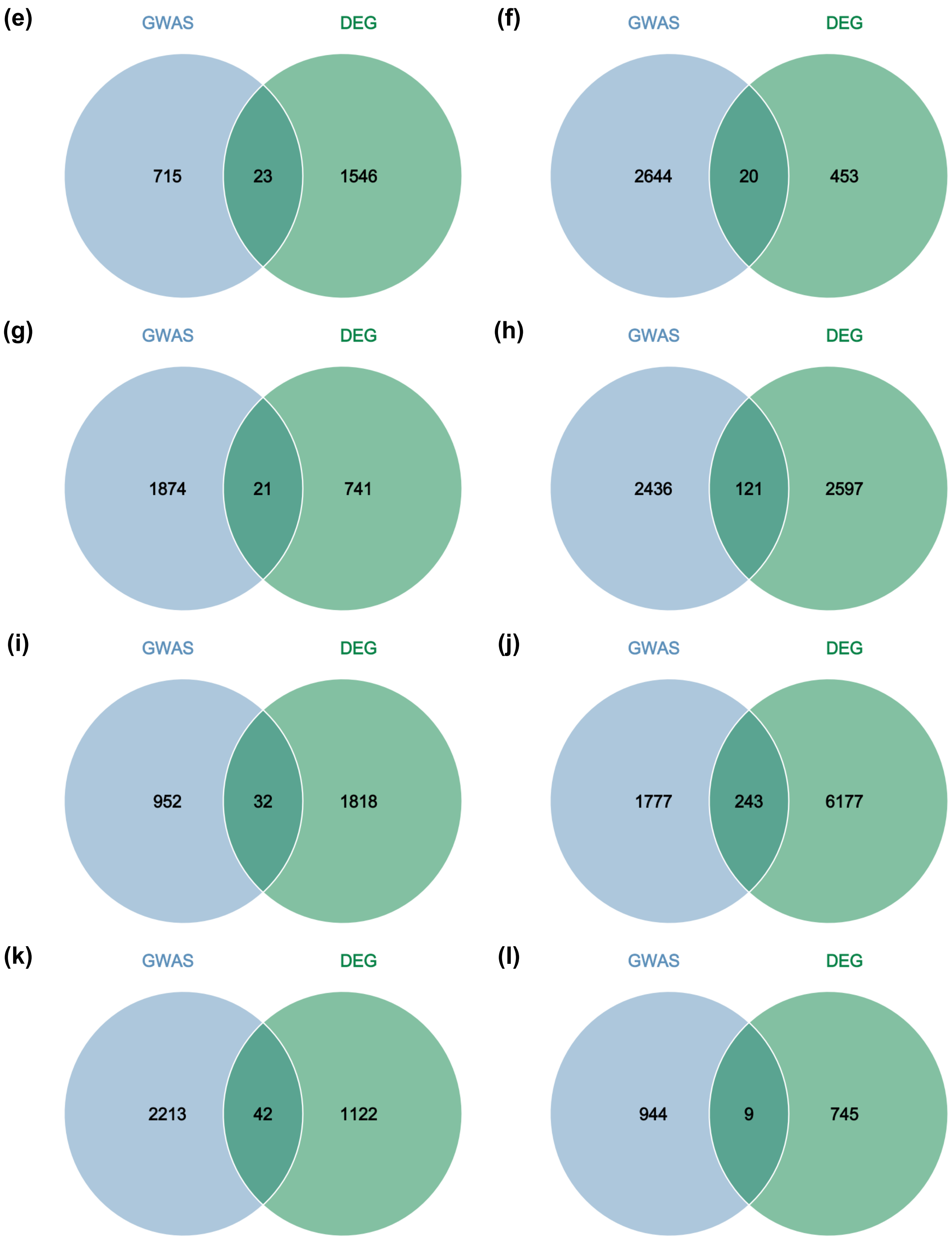
(e)
GWAS
DEG
715
23
1546
(f)
GWAS
DEG
2644
20
453
(g)
GWAS
DEG
1874
21
741
(h)
GWAS
DEG
2436
121
2597
(i)
GWAS
DEG
952
32
1818
(j)
GWAS
DEG
1777
243
6177
(k)
GWAS
DEG
2213
42
1122
(l)
GWAS
DEG
944
9
745

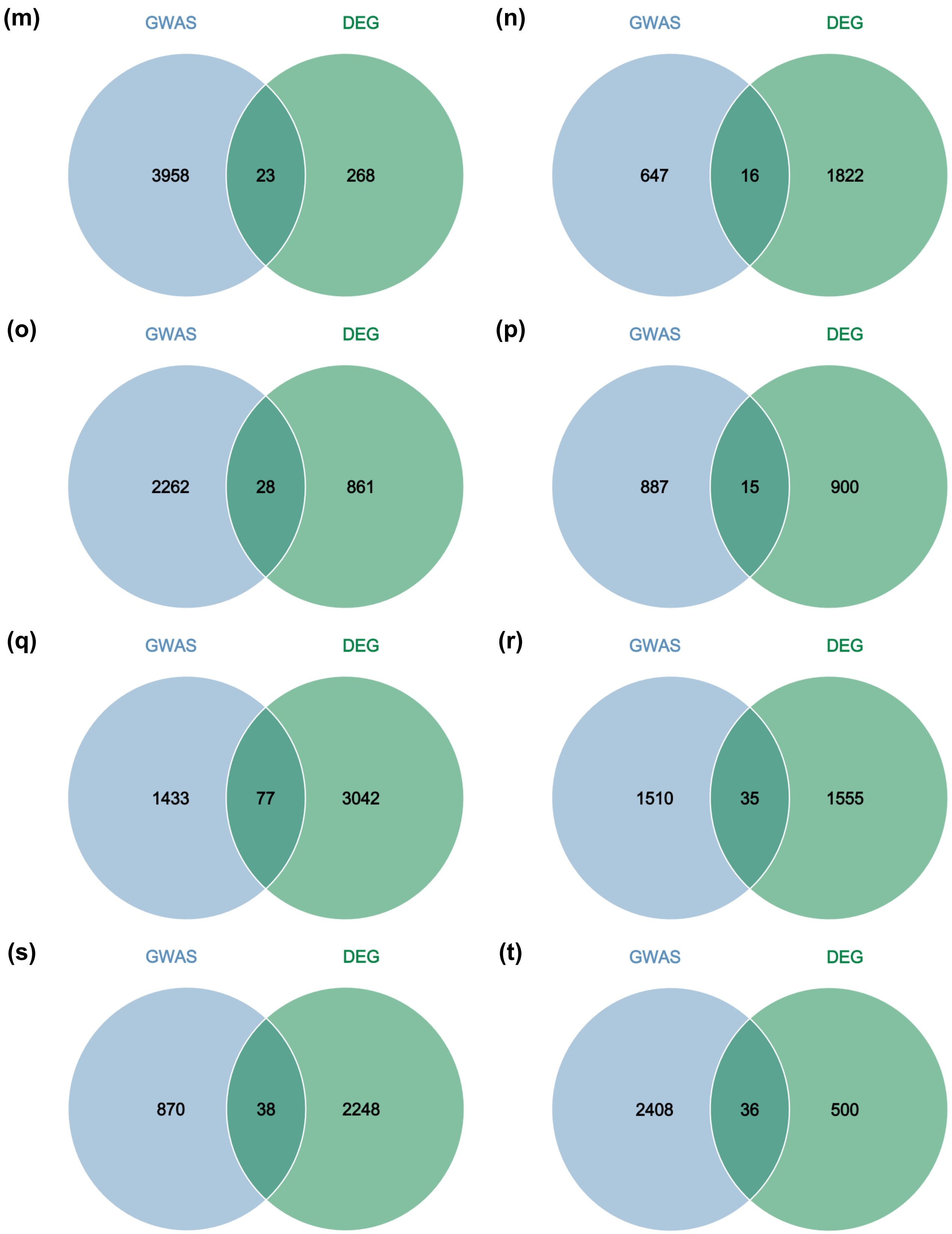
(m)
GWAS
DEG
3958
23
268
(n)
GWAS
DEG
647
16
1822
(o)
GWAS
DEG
2262
28
861
(p)
GWAS
DEG
887
15
900
(q)
GWAS
DEG
1433
77
3042
(r)
GWAS
DEG
1510
35
1555
(s)
GWAS
DEG
870
38
2248
(t)
GWAS
DEG
2408
36
500

(u)

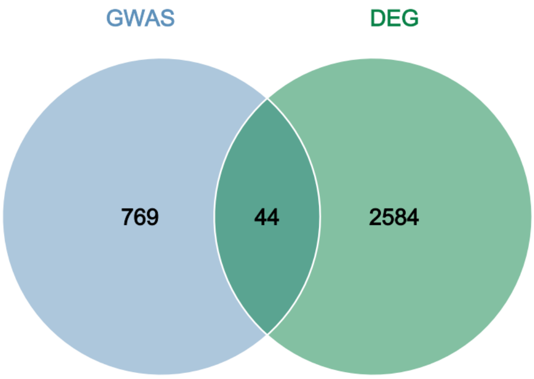

(a):Ala; (b):Arg; (c):Asp; (d):Cys; (e):Glu; (f):Gly; (g):His; (h):Ile; (i):Leu; (j):Lys; (k):Met; (l):Phe; (m):Pro; (n):Ser; (o):Thr; (p):Tyr; (q):Val; (r):Bitter; (s):TAA; (t):Sweet; (u):Umami;
